# Cyclic Analogs of Desferrioxamine E Siderophore for ^68^Ga Nuclear Imaging: Coordination Chemistry and Biological Activity in *Staphylococcus aureus*

**DOI:** 10.1021/acs.inorgchem.1c02453

**Published:** 2021-11-16

**Authors:** Andrzej Mular, Abraham Shanzer, Henryk Kozłowski, Isabella Hubmann, Matthias Misslinger, Julia Krzywik, Clemens Decristoforo, Elzbieta Gumienna-Kontecka

**Affiliations:** † Faculty of Chemistry, University of Wrocław, 50-383 Wrocław, Poland; ‡ Department of Organic Chemistry, The Weizmann Institute of Science, Rehovot 7610001, Israel; § Department of Health Sciences, University of Opole, 45-060 Opole, Poland; ∥ Department of Nuclear Medicine, Medical University Innsbruck, A-6020 Innsbruck, Austria; ⊥ Institute of Molecular Biology, Medical University Innsbruck, A-6020 Innsbruck, Austria; # TriMen Chemicals, Piłsudskiego 141, 92-318 Łódź, Poland

## Abstract

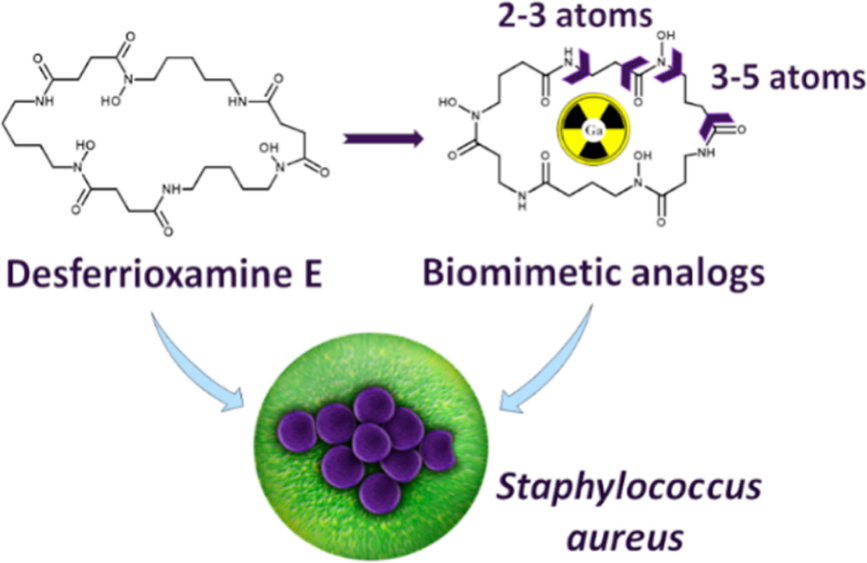

As multidrug-resistant bacteria are an emerging problem and threat to humanity, novel strategies for treatment and diagnostics are actively sought. We aim to utilize siderophores, iron-specific strong chelating agents produced by microbes, as gallium ion carriers for diagnosis, applying that Fe(III) can be successfully replaced by Ga(III) without losing biological properties of the investigated complex, which allows molecular imaging by positron emission tomography (PET). Here, we report synthesis, full solution chemistry, thermodynamic characterization, and the preliminary biological evaluation of biomimetic derivatives (FOX) of desferrioxamine E (FOXE) siderophore, radiolabeled with ^68^Ga for possible applications in PET imaging of *S. aureus*. From a series of six biomimetic analogs, which differ from FOXE with cycle length and position of hydroxamic and amide groups, the highest Fe(III) and Ga(III) stability was determined for the most FOXE alike compounds–FOX 2-4 and FOX 2-5; we have also established the stability constant of the Ga-FOXE complex. For this purpose, spectroscopic and potentiometric titrations, together with the Fe(III)–Ga(III) competition method, were used. [^68^Ga]Ga-FOXE derivatives uptake and microbial growth promotion studies conducted on *S. aureus* were efficient for compounds with a larger cavity, i.e., FOX 2-5, 2-6, and 3-5. Even though showing low uptake values, Fe-FOX 2-4 seems to be also a good Fe-source to support the growth of *S. aureus*. Overall, proposed derivatives may hold potential as inert and stable carrier agents for radioactive Ga(III) ions for diagnostic medical applications or interesting starting compounds for further modifications.

## Abstract 2

In this work, the authors have investigated a set of novel ferrioxamine E analogs as potential Ga-68 chelators and tools for infection imaging.

## Introduction

Bacterial infectious diseases remain a major threat to humanity. Development of multidrug resistant bacteria caused by misuse of antimicrobial agents in medicine and agriculture, global travel, and rapid spread of diseases, as well as the growing number of medical procedures, has caused one of the most pressing global concerns of antibiotic resistance, and formerly routine therapies are becoming challenging.^1^ The situation is most severe in patients whose immune systems are compromised, for example, during chemotherapy or transplantation. Multidrug-resistant microbes are a rising problem, and hospital-acquired infections (HAIs) caused by opportunistic pathogens are one of the main public health challenges in developed countries.^2^

Timely and precise detection and localization of infection are critical for effective medical intervention and proper treatment. Microscopy, microbiology, and molecular techniques are the most common diagnostic tools, but all have their drawbacks, since they depend on the availability of relevant clinical samples. Mostly it is urine or blood which can lack relevance, especially in the case of a deep-seated or undeveloped infection. Biopsies as a direct intervention into organisms are also burdened with risk and susceptible to insensitive sampling restricted to the small area of a biopsy needle. Finally, the time required for microbial culture growth is considerably long (48–72 h) and in the case of patients in harsh conditions often results in severe complications including death.^2,3^

Novel strategies for diagnosis and treatment are actively sought. Alternative diagnostic methods are implemented in rigid infection treatments including those based on radiotracers: PET and SPECT. Molecular imaging approaches do not require sample collection and are able to detect biological and biochemical alterations at the infection site from the earliest stages of the disease.^2^ A significant number of nonspecific radiolabeled tracers are currently in use,^4,5^ but they mostly target secondary symptoms of disease including the following: increased blood flow and vascular permeability, activated endothelial cells, or polymorphonuclear cell migration.

In an attempt to find more specific radiotracers, an interesting group of naturally occurring compounds came to light. Siderophores are low molecular mass chelators which are used by microbes for iron sequestration and are considered as virulence factors during infections.^6^ Siderophore systems require advanced recognition and transport mechanisms. In bacteria, siderophore internalization is typically operated by ABC type transporters, with some exceptions when iron-loaded siderophores are translocated by inner membrane permeases driven by proton motive force. In Gram-negative bacteria, the iron-loaded siderophore is first recognized and internalized by specific outer membrane receptors (supplied by a TonB complex, which provides the energy through the proton motive force). These highly sophisticated machineries are not present in mammalian cells.^7−9^ Therefore, the microbial Fe(III) transport system seems to be a perfect target for specific diagnostic applications and therapeutic agents with selective toxicity. Although Fe(III) does not possess an isotope suitable for nuclear imaging, Ga(III) can successfully mimic Fe(III) in its complexes because of its similarity in coordination properties. Ga(III) is characterized by an equal charge and a similar radius to Fe(III) enabling the displacement of iron by gallium in its complexes. Ga(III) is considered as an isosteric and diamagnetic surrogate for Fe(III) and was excessively used in siderophore characterization^10^ and for detecting opportunistic respiratory tract infections.^11,12^ During the past decade, the ^68^Ga isotope was intensively investigated as a radiolabeling agent since PET scans became a standard in clinics, and novel, more accessible radioisotopes were actively sought. ^68^Ga exhibits very convenient decay properties with a half-life of 68 min, prevalent β^+^ decay yield (88%, 1899 keV),^13^ convenient for nuclear imaging with small molecules and peptides,^14^ and causes very low side effects since emitted radiation is low. Moreover, this isotope can be obtained from ^68^Ge/^68^Ga generators which are portable, accessible, and simple in use.^15,16^ A siderophore-based “Trojan Horse” strategy using Ga(III)–siderophore complexes reveals promising clinical relevance.^17−22^

FOXE (known as nocardamine) is one of the strongest among the hydroxamate siderophores. This cyclic chelator is built of alternating diamine and dicarboxylic acid building blocks connected by amide bonds and was first isolated from a *Streptomyces sp.*(23) and then found in other bacterial species: *Pantoea agglomerans*,^24^*Pseudomonas stutzeri*,^25^ and *Hafnia alvei*.^26^ Although native siderophores are usually not species-specific, show broad-spectrum activity, and are recognized by several types of microorganisms,^27^ bacteria have been shown to produce various macrocyclic ferrioxamines in response to cultivation conditions^28^ and in feeding studies, depending on available precursors.^29,30^ Siderophores were found to be produced by all microbes in four different siderophore-related social interactions. Microbes can share the same siderophore during uptake among clonal cells, they can steal siderophores if they share the same uptake receptor, competition by locking away may occur, and by metabolite competition microbes strive which chelator will be most efficient and cost-effective to sequester available iron ions from their niche.^27^ Of importance, this adaptive phenomenon was shown to be used by *Streptomyces* sp. to prevent fungal infections by starving the fungi of Fe(III) via overproduction of new analogs of desferrioxamines, both linear and cyclic.^31^

*Staphylococcus aureus* is a leading causative agent of bacterial infections in humans. In the case of multidrug resistant strains (MRSA) like methicillin-resistant *S. aureus*, the infection can develop into lethal, incurable diseases, especially in immunocompromised patients or in the course of HAI. *S. aureus* holds second place as the most common cause of bacteremia which can rapidly develop into sepsis. The mortality of *S. aureus* bacteremia is high, 15–50% despite implemented treatment, and varies depending on the world region. The essential part of the therapy is to localize and control the source of infection.^2,32,33^*S. aureus* expresses several virulence factors, and siderophores are one of the most prominent ones. Several independent experiments demonstrated that *S. aureus* is able to produce three different iron chelators: staphyloferrin A,^34,35^ staphyloferrin B,^36,37^ and aurochelin^38^ but is not capable of producing any hydroxamate-type siderophores; nevertheless, it is able to utilize them for enhanced growth under iron restricted conditions thanks to ATP-binding cassette transporters (ABC) dependent, ferric hydroxamate uptake (Fhu) system.^39,40^ Recognition and utilization of xenosiderophores (siderophores that are not self-origin) is one of the mechanisms applied by bacteria and fungi during niche competition for iron.^41^

In this work, six derivatives of the natural siderophore FOXE were examined as potential Ga carriers for therapeutics and diagnosis. We have established full solution chemistry of FOXE analogs, and ^68^Ga radiolabeled siderophores were preliminarily evaluated with respect to recognition by *S. aureus*.

## Results and Discussion

### Ligands’ Design and Synthesis

**Scheme 1:**
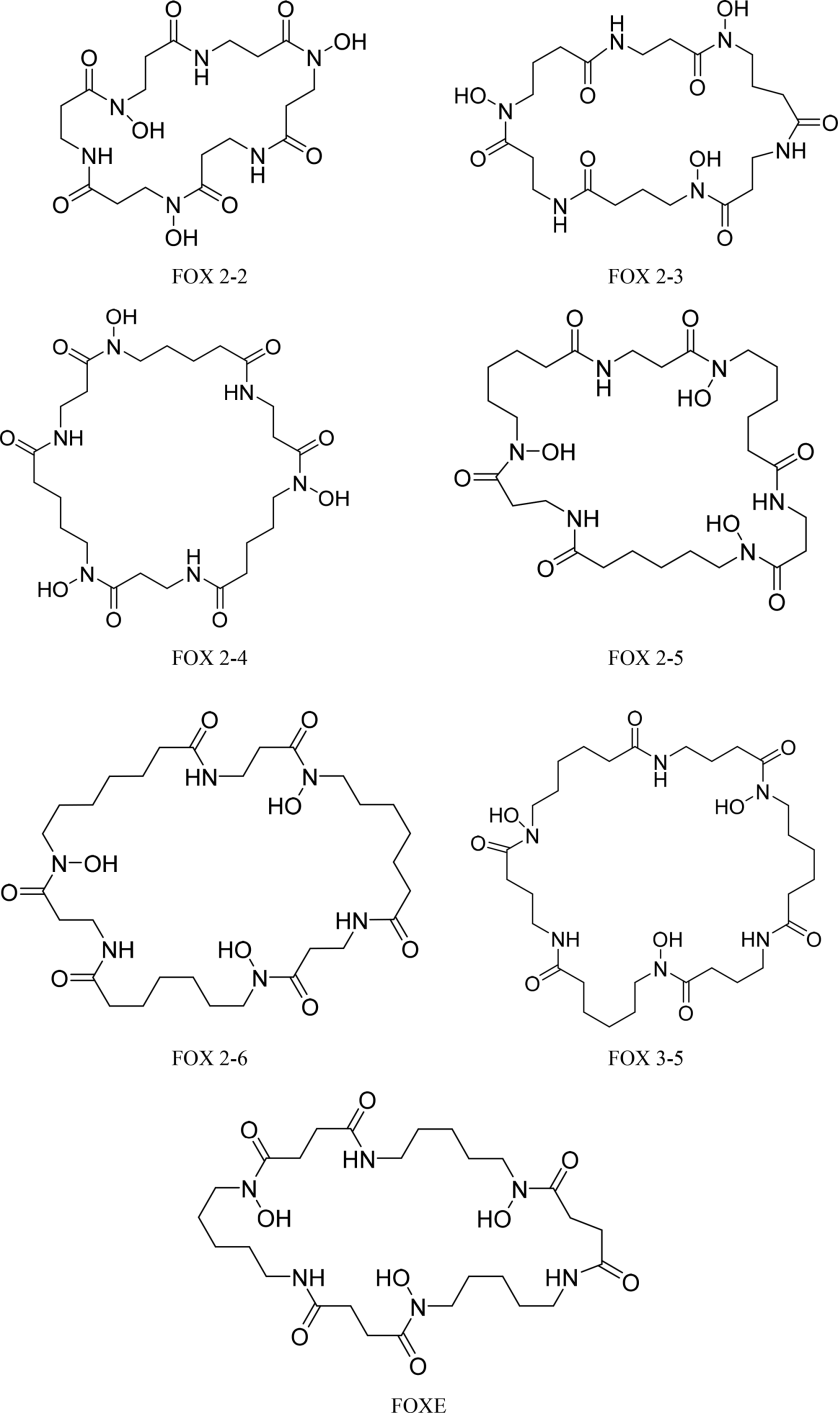
Structures of FOXE and Its Analogs Investigated in This Work

An investigated series of FOXE analogs consists of six novel derivative compounds. Detailed structures of all the analogs in this series are provided in Scheme 1, together with the structure of natural FOXE, possessing a 33-membered ring with three evenly spaced hydroxamic groups. The iron binding cavities in the designed biomimetic compounds were modified systematically and differ from the naturally occurring siderophore FOXE with cycle length and position of the hydroxamic group in relation to the amide group. Five analogs have the same ethyl spacing group between amide and hydroxamic groups when FOX 3-5 has a propyl group spacing them. In the FOXE analogs FOX 2-2, FOX 2-3, FOX 2-4, FOX 2-5, FOX 2-6, and FOX 3-5, the numbers determine the carbon atom quantity between the hydroxamic–amide–hydroxamic groups, with the hydroxamic acids positioned retro in relation to natural FOXE, −CON(OH)– and −N(OH)CO–, respectively (Scheme 1). FOX 2-6 and FOX 3-5 are of the same composition and molecular mass, but they differ in binding groups constitution. This small alteration has influenced greatly hydrophilicity (log P, *vide infra*) of the compound to an extent that FOX 2-6 was not water soluble in concentrations used during our assays, and we were not able to determine solution chemistry for this analog. However, this change seems not to influence microbial recognition in a significant manner.

Reversing the order of hydroxamic groups does not influence their coordination characteristics.^42^ First, synthesis of FOXE retro derivatives was performed by Shimizu et al.^43^ and was described as not demanding. Indeed, synthesis of retro analogs of desferrioxamines is more convenient, and products are more stable.^44,45^ For this reason, we decided to implement changes in the compound constitution. Moreover, we wanted to examine if differences in molecules’ cycle size can alter coordination properties of FOXE analogs and if these structurally changed moieties would still be recognized by microbes, thereby acting as siderophores.

As purification and crystallization of siderophore receptors are highly demanding, applied structural changes may indicate which regions of the siderophore moiety are crucial for microbial recognition and which positions can be altered, for instance, during future bioconjugate synthesis. In general, native siderophores lack suitable sites for incorporating additional functionalities; e.g., fluorescent probes, surface-adhesive moieties, or drug molecules, with applications in imaging and/or as therapeutic conjugates trafficked into microbial species via the siderophore recognition system.^46,47^ Those limitations can be overpassed by specially designed biomitetic analogs which could become novel tools for both diagnostics and therapeutics.^44,48,49^ Additionally, synthetic biomimetic analogs offer the advantage of being more easily translated into a clinical setting, allowing a more standardized, reproducible, and cost-effective production in compliance with Good Manufacturing Practices (GMP) as compared to fermentation processes for natural siderophores.

**Scheme 2:**
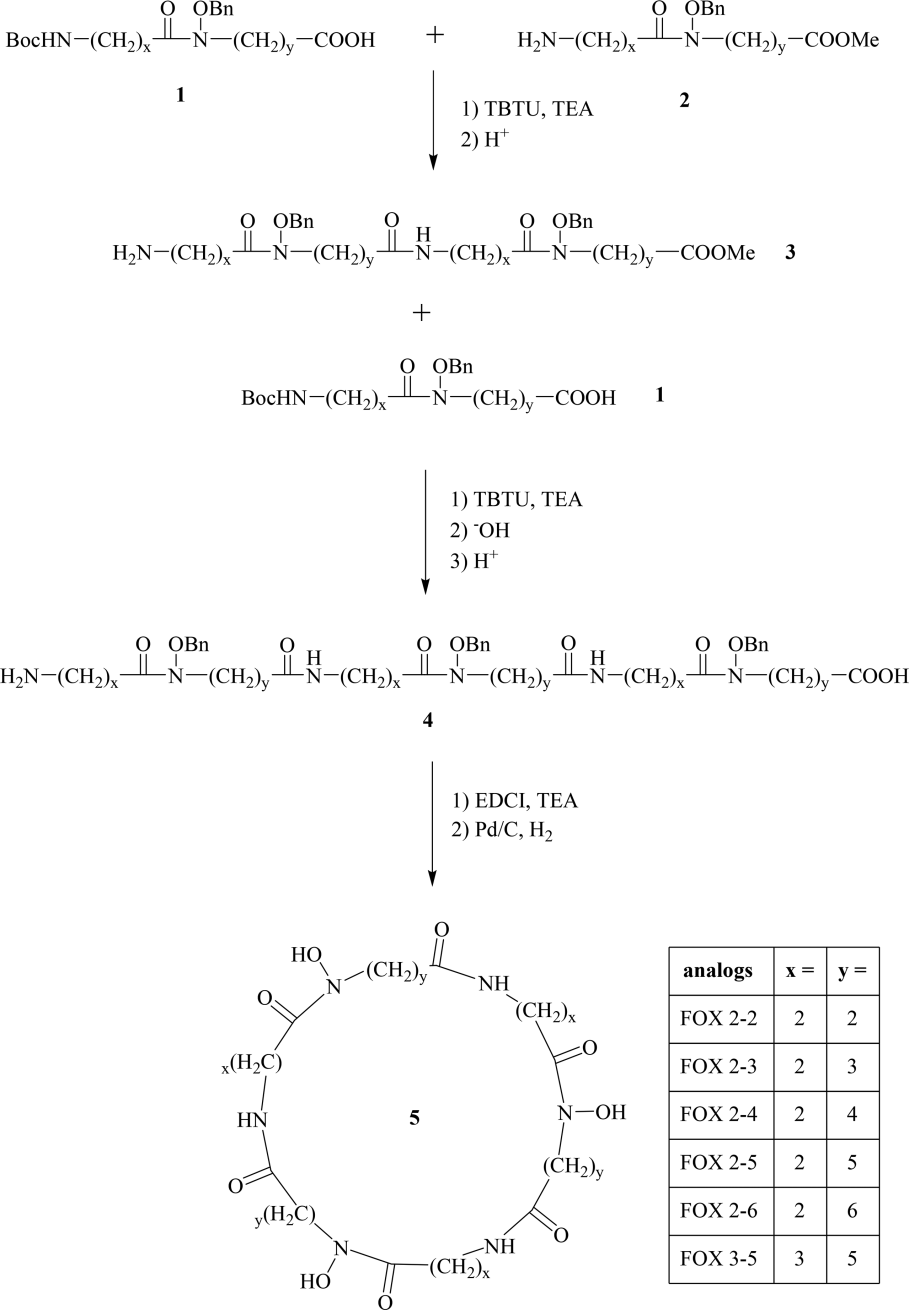
General Route for the Synthesis of FOXE Analogs

All the designed compounds were synthesized commercially by TriMen Chemicals (Łódź, Poland) and used as received. The synthetic strategy is illustrated in Scheme 2. In order to carry out the designed transformations, it was necessary to use orthogonal protecting groups: the amine function was blocked with the *tert*-butyloxycarbonyl group (Boc), the carboxyl function was blocked with a methyl or ethyl ester (Me or Et), and the hydroxyl group in the hydroxylamine fragment was blocked with the benzyl group (Bn).

The first step of synthesis involved the coupling of fragments **1** and **2**, which was performed with TBTU (*O*-(benzotriazol-1-yl)-*N,N,N′,N′-*tetramethyluronium tetrafluoroborate) with the addition of TEA (triethylamine). The amine group was deprotected with acid to give the intermediate **3**. Compound **3** was then linked to compound **1** with TBTU, and according to the information provided in Scheme 2, the blocking groups from the carboxyl and amine functions were removed to give compound **4**. Intermediate **4** was then cyclized to form an amide bond using EDCI (*N*-(3-dimethylaminopropyl)-*N′*-ethylcarbodiimide) as a coupling reagent. The last step involved removing the benzyl moiety from the hydroxyl groups with hydrogen in the presence of Pd/C to give **5**. The purity and structures of the obtained ligands (FOX 2-2, FOX 2-3, FOX 2-4, FOX 2-5, FOX 2-6, FOX 3-5) were determined using ^1^H NMR and ESI-MS methods and are shown in the Supporting Information (Figure S1 and Table S1) together with the detailed description of the synthetic procedures. For further physicochemical studies, the purity was checked potentiometrically.

### Thermodynamic Solution Studies

Applicable radiopharmaceuticals should be characterized by high stability *in vivo* and kinetic inertness. Efficacious biomimetics must accomplish the role of the natural compound they mimic. Siderophores not only supply iron to microbial cells but also bind the ferric ion with extraordinary affinity in order to overpass cross-chelation and sequester iron ions from host proteins.^50^ To determine if novel FOXE analogs will be stable enough in the pH range of human serum and will withstand cross-chelation with ligands present in living organisms, it is necessary to define the coordination properties of examined compounds.

Elaborated solution thermodynamic studies combining several methods were performed in order to investigate those properties. Five FOXE analogs were water soluble in at least 1 × 10^–3^ M concentration. FOX 2-6 was not soluble in H_2_O nor in a H_2_O/DMSO 70/30 w/w mixture, and its thermodynamic properties were not examined. Major results of physicochemical analysis for analogs characterized by most desirable properties are summarized below. For the rest of the data and full details leading to these results, refer to the Supporting Information.

In the first step, stoichiometry of forming complexes was determined using ESI-MS. Next, to examine if structural changes to the FOXE structure influenced strong metal binding abilities, ligands’ acid–base properties and Fe(III)-FOXE analogs formation constants were studied; potentiometric and spectrophotometric titrations were used to characterize forming complexes in wide pH range. In addition, to confirm previously calculated stability constants, ligand–ligand competition titrations of Fe(III) complexes vs EDTA were conducted. Finally, Ga(III) complexes were characterized, and their formation constants were determined in Fe(III)–Ga(III) metal–metal competition experiments. Additionally, from CV assays, we have determined the standard redox potential of Fe(III) complex species, and by refining this data, we have delivered stability constants for Fe(II) complexes with FOXE derivatives. From combining this information, full coordination characteristics of the studied series of analogs and their Fe(III) and Ga(III) complexes were obtained.

#### The Equilibria of Fe(III) and Ga(III) Complexes Formation

Electrospray mass spectrometry (ESI-MS) is widely used for characterization of metallic complexes in solution.^51^ Regardless of the fact that this method cannot distinguish the ionizable protons in the forming species, it can be used to deliver accurate information about metal-to-ligand stoichiometry. The *m*/*z* values obtained in MS experiments were successfully employed by our research group on numerous occasions.^52,53^ A few major peaks characterized the MS spectra of the investigated solutions of the Fe(ClO_4_)_3_/ligand and Ga(ClO_4_)_3_/ligand with a 1:1 ratio (Figure S2). The peaks were attributed to the mononuclear species (Table S2, Table S3), and no peaks corresponding to the free ligand were present.

The formation of Fe(III)-FOX complexes was demonstrated by spectroscopic evolution of the LMCT band with its maximum moving from ∼470 to 430 nm after pH was raised from ∼0.1 until ∼6.5–8.9 (Figure S3). At a very acidic pH, the presented maximum corresponded to the formation of dihydroxamate ferric complexes, while from pH around 3, it switched gradually to 430 nm which is characteristic of trihydroxamate Fe(III) complexes, suggesting two species in equilibrium over the whole pH range, [FeHL]^+^ and [FeL]. In the case of the Fe(III)-FOX 2-5 system, the UV–vis spectra were however different; only one band was present over the whole titration (Figure S3g) leading to the conclusion that only the fully coordinated form of the Fe(III)-FOX 2-5 complex, [FeL], was present. The overall stability constants, log*K*_FeL_, are given in Table 1, and full data are presented in the SI (Table S5, Table S6). Speciation plots are presented in Figure S7. In order to confirm the stability constants of the trihydroxamate cyclic forms of discussed ferric complexes, the spectrophotometric competition experiments with EDTA were performed at fixed pH 7.0 (Figure S4). The logβ_FeL_ values (Table S7) were calculated according to eqs S6 and S7 and using known values of protonation constants of respective ligands (Table S4), EDTA, and stability constants of Fe(III)-EDTA.^54^

**Table 1:** Overall Stability Constants (logβ_ML_) for Studied Complexes with Fe(III), Ga(III), and Fe(II) Ions, pH Independent Redox Potential (*E*_1/2_ vs NHE), and pFe(III) and pGa(III) Values

desferrioxamine	logβ_Fe(III)L_^a^	logβ_Ga(III)L_^a^	logβ_Fe(II)_^b^	*E*_1/2_ [mv] vs NHE^b^	pFe(III)^e^	pGa(III)^f^
FOX 2-2	25.92(8)	26.44(5)	7.48	–369	21.5	21.4
FOX 2-3	27.22(2)	25.14(9)	7.54	–432	22.7	20.8
FOX 2-4	28.71(7)	26.29(7)	9.12	–426	24.3	21.8
FOX 2-5	31.32(8)	29.50(6)	11.29	–452	27.0	25.2
FOX 3-5	28.81(2)	27.17(4)	8.48	–416	24.1	22.6
FOXE	32.21(4)^c^	29.79(1)	12.1^d^	–477^d^	27.3^c^	25.2
DFOB	31.10^g^	27.56^g^			26.3^f^	21.6^g^
transferrin					25.6^h^	20.3^i^

a Constants determined from potentiometric and UV–vis pH-dependent titrations. Conditions: [L] = 1 × 10^–3^ M, M:L 1:1 for potentiometric assays and 5 × 10^–5^ M, M:L 1:1 for UV–vis assays, *T* = 25 °C, *I* = 0.1 M NaClO_4_.

b Constants determined from CV measurement and calculated for the NHE electrode. Conditions: [L] = 2 × 10^–3^ M, M:L 1:1, pH = 7, *T* = 25 °C.

c Reference (30).

d Reference (61).

e Calculated for conditions: [L] = 1 × 10^–4^ [M] = 1 × 10^–5^ at pH 7.4.

f Reference (63).

g Reference (58).

h Reference (64).

i Reference (65).

**Figure 1:**
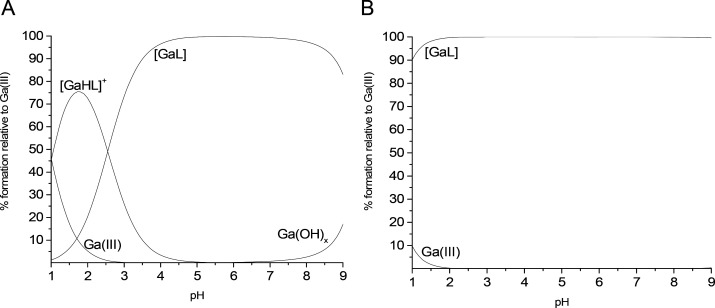
Speciation plots of Ga(III)-FOX 2-4 (A) and Ga(III)-FOX 2-5 (B) complexes calculated with protonation and stability constants from Table S4 and Table S8. Calculated for conditions: [L] = 1 × 10^–3^ M, [Ga(III)] = 1 × 10^–3^ M in the pH range 1–9.

The next step was to determine the coordination specificity of investigated ligands toward Ga(III) ions. This is a somewhat challenging task because it requires indirect methods since the investigated complexes started to form in a very acidic environment, where potentiometric measurements were not accurate owing to the error of the glass electrode,^55,56^ and gallium is spectroscopically silent in UV–vis. To bypass this inconvenience, we implemented metal–metal competition assays. After addition of a significant molar excess of Ga(III), the LMCT band of Fe(III)-FOXE analog complexes was almost fully silenced as Ga(III) replaced Fe(III) in its complexes (Figure S5). By implementing those spectral changes, we were able to calculate the stability constants of the Ga(III)-FOXE analog species present below or around pH 2, [GaHL]^+^ (Table S8). Stability constants for the [GaL] species, present above pH 2, could be determined by potentiometric titrations for Ga(III)-FOX 2-2, 2-3, 2-4, and 3-5, with logβ of [GaHL]^+^ fixed in the calculations (Table S8). For Ga(III)-FOX 2-5, we could not observe any deprotonation step using this methodology. To confirm this behavior, UV–vis titration of the Ga(III)-FOX 2-5 analog complex was performed in the 200–300 nm spectral range (Figure S6) corresponding to the hydroxamate groups’ protonation state.^57−59^ Titration performed within the pH range 0.1–4 showed a gradual rise of the band with λ_max_ at 225 nm attributed to ligand deprotonation and coordination of Ga(III) ions (p*K*_NHOH_ = 0.76). Above pH 4, there were no significant changes in maxima localization or absorbance intensity. Above pH 9, absorbance started to rise again, which indicated complex dissociation. Therefore, from this particular assay, we could state that Ga(III)-FOX 2-5 is stable in the pH range of human serum. Overall, the data indicated a decreased stability of [GaL] species when decreasing or increasing the ring size of Ga(III)-FOX 2-5, as reflected by p*K*_NHOH_ (Table S8). Implementing this full coordination specification of Ga(III)-FOX complexes, we were able to calculate speciation plots which are presented in Figure 1 for the most efficient analogs FOX 2-4 and FOX 2-5, given here as examples, and in Figure S7 for others.

On the pH-dependent distribution plots of the FOX 2-4 analog (Figure 1A), one can observe that the [GaHL]^+^ species is formed already at pH 1 and reaches its maximum concentration around pH 1.5. The [GaL] complex, formed with p*K* 2.55, is a dominant species slightly above pH 3, reaching its maximum concentration around pH 4, and dominates in the solution to pH around 8.5 where monomeric hydroxide species of Ga(III) start to be present. In the case of the Ga(III)-FOX 2-5 analog complex (Figure 1B), the [GaL] form is a dominant species along the whole pH range studied.

#### Complex Formation Equilibria with Fe(II)

Redox chemistry of Fe(III) species was determined in cyclic voltammetry (CV) assays. This data is of biological significance, as it reflects the possibility of a reductive mechanism of Fe release within microbial cells utilizing specific siderophores for ferric ion acquisition.^60^ We have obtained quasireversible voltammograms for Fe(III)-FOXE analog complexes (Figure S8), with Δ*E* ranging from −369 mV to −470 mV. The values of *E*_1/2_ (Table 1) were recalculated vs normal hydrogen electrode (NHE). To ensure that only fully hexacoordinated complexes [FeL] were characterized, experiments were carried out at fixed pH = 7 where this form is dominating in solution. Higher strength of the complex (larger pM value) correlates to lower redox potential. This behavior was in agreement with previously reported data^61,62^ for similar complexation systems.

Overall, in comparison with gathered CV data^61^ for Fe(III)/Fe(II)-FOX complexes, the Fe(III) complexes of studied ligands showed a less negative redox potential, revealing their lower stability (*vide supra*). The estimated binding constants of Fe(II)L complexes, calculated with eq S1, were decreased by around 20 orders of magnitude in relation to stability constants of corresponding Fe(III)L complexes (Table 1), which is typical for hydroxamate siderophores.^61^ This can be explained by the preference of oxygen hard donor atoms for ferric Fe(III) cations over softer ferrous Fe(II) cations.

#### pM of Investigated FOXE Analog Complexes

Overall formation constant (logβ) allows the measurement of the strength of the metal–ligand interaction and the thermodynamic basis for the specificity of metal ion, yet its use could be problematic to compare, especially if few complex species are present at the same pH or compared ligands differ in denticity. Therefore, a better and more intuitive measure for comparison with other ligands would be the pM value, defined as *pM* = −log[*M*]_*n*+_^*free*^, where [*M*]_*n*+_^*free*^ is the concentration of free metal ion at pH 7.4, with a total ligand concentration of 10^–5^ M and total metal concentration of 10^–6^ M.^64,66^ The pM value focuses on free metal ion concentration rather than ligand–metal interactions. Higher pM values indicate a less unbound metal ion in the solution and consequently stronger binding ability of the corresponding ligand. Collected data (Table 1) show that pM values of investigated ligands stay within the range of the natural siderophore FOXE, and the most promising of investigated complexes, Ga(III)-FOX 2-5, is weaker only by 0.7 orders of magnitude than its natural analog FOXE. Comparing pM values, we can notice a pattern where the most FOXE alike ligand, FOX 2-5, is characterized by the highest chelating efficiency, and any alteration in internal cavity size causes worsening of metal binding properties.

From these combined data, we can suggest a coordination model. Starting at a strongly acidic pH of 0.1, two hydroxamate residues are already coordinated to the corresponding metal ion resulting in [FeLH]^+^ species. At higher pH, after deprotonation of the last hydroxamate group, the fully coordinated complex of [FeL] is formed where three hydroxamates are participating in chelation resulting in a hexacoordinate complex with octahedral geometry. For the strongest ligand in the series, FOX 2-5, only the fully coordinated form is present from pH 0.1. Only 1:1 stoichiometry complexes are forming since the cyclic structure sterically forces this coordination model.

All investigated complexes are characterized by exceptional stability constants attributed to similar siderophore systems (Table 1). These highly promising properties arise from the fact that our compounds were designed specifically for Fe(III) chelation. Ligands with six oxygen donor atoms (hard base atoms according to Pearson’s principle) are the perfect match for full saturation of the hexacoordinate coordination sites of the Fe(III) ion (hard acid atoms). The cyclic structure of our ligands is an additional asset due to the chelation effect which further enhances coordination properties and kinetic inertness which not only results in high stability constants but also prevents cross-chelation in the presence of other strong ligands such as transferrin. As Ga(III) exhibits almost identical coordination properties as Fe(III), its complexes are highly alike. Both Fe(III) and Ga(III) complexes are characterized by lower stability constants than natural siderophores. The highest stability is attributed to the most FOXE alike compounds–FOX 2-4 and FOX 2-5. From this pattern, we can arrange the investigated analogs in a series, from weakest Fe(III) and Ga(III) chelators to the strongest:

FOX 2-2 < FOX 2-3 < FOX 3-5 < FOX 2-4 < FOX 2-5 < FOXE

Moreover, this pattern was exhibited for ferrous Fe(II) complexes as well.

From the collected data, we can state that the best coordination properties toward Fe(III) and Ga(III) cations are exhibited by ligand FOX 2-5 with a 33-membered cycle ring and ligand FOX 2-4 with a 29-membered cycle ring. It seems that the interior cavity of this size perfectly suits Fe(III) ions. As expected, Ga(III) cations exhibit similar coordination properties, but their complexes are weaker by approximately 2 orders of magnitude than ferric ones. Overall, we can deduce that the alteration of cycle size of studied ligands negatively influences coordination properties toward iron and gallium ions in comparison to the natural siderophore FOXE. Expanding or reducing the size of the interior cavity undoubtedly worsens coordination properties toward the investigated metal ions. As for other structural changes, the retro position of the hydroxamate groups seems not to influence chelation strength toward Fe(III) ions in a significant manner as the FOX 2-5 analog presents only a slightly lower (logβ_Fe(III)L_ = 31.32) stability constant than FOXE (logβ_Fe(III)L_ = 32.21),^30^ confirming again what has been shown previously.

#### Characterization of ^68^Ga-Labeled FOXE Analogs

All ligands could be labeled with gallium-68 using 10 μg of the ligand at high molar radioactivity of 2-6 GBq/μmol. Under these conditions only with FOX 2-5 quantitative labeling yields as determined by HPLC (99.3 ± 0.4, *n* = 5) and TLC (99.1 ± 0.9, *n* = 5) were achieved in all cases; [^68^Ga]Ga-FOX 2-2, 2-3, 2-4, 2-6, and 3-5 showed variable radiolabeling yields, if below 95%, as determined by TLC, and they were purified by SPE before further use. Table S10 summarizes radiochemical purity (RCP) results as determined by different methods. For [^68^Ga]Ga-FOX 2-2, 2-3, and 2-4 in particular, results from TLC were considerably lower as compared to RCP calculated from Sep-Pak purification. This could be explained by partial decomposition of the formed complexes in the high concentrated citrate solution used as solvent for TLC; in the case of [^68^Ga]Ga-FOX 3-5, all activity migrated with the solvent front, making evaluation of RCP by TLC impossible. Values from HPLC were also lower as compared to SPE, indicating instability of these [^68^Ga]Ga-FOX complexes in the HPLC analyte. Overall, for further optimization of radiolabeling procedures, also analytical methods need to be further optimized, except for [^68^Ga]Ga-FOX 2-5, where all analytical methods correlated very well and quantitative labeling is easily achieved.

logD values ranged from −3.0 to −0.57, as listed in Table 2. Although smaller FOXE derivatives generally showed higher hydrophilicity than larger compounds, distribution coefficients did not strictly correlate with the length of carbon chains or the molecular weight, as seen in the difference of logD values of FOX 2-2, FOX 2-3, and FOX 2-4. Retention times on HPLC are also shown in Table 2, overall, with a corresponding trend as seen in logD values with higher retention times for longer carbon chains. Retention times of metal complexes were overall shorter than for the free ligand, and Fe- and ^68^Ga-complexes showed corresponding retention times except for FOX 2-2 possibly indicating incomplete coordination of the ^68^Ga-FOX 2-2 complex under the acidic HPLC-assay conditions. Representative HPLC and TLC chromatograms for FOX 2-5 are shown in Figure S10.

**Table 2:** logD Values of ^68^Ga-Labeled FOX Derivatives (*n* > 6)

	logD	SD	HPLC-RtFOX	HPLC-RtFe-FOX	HPLC-Rt[^68^Ga]Ga-FOX
FOX 2-2	–2.66	0.03	10.1	10.4	9.7
FOX 2-3	–2.96	0.01	11.0	10.3	10.3
FOX 2-4	–3.02	0.03	11.4	10.5	10.5
FOX 2-5	–1.90	0.03	12.3	11.8	11.7
FOX 2-6	–0.58	0.01	13.4	13.0	13.0
FOX 3-5	–1.57	0.00	12.8	12.0	12.0

#### Biological Characterization in *S. aureus* Cultures

**Figure 2:**
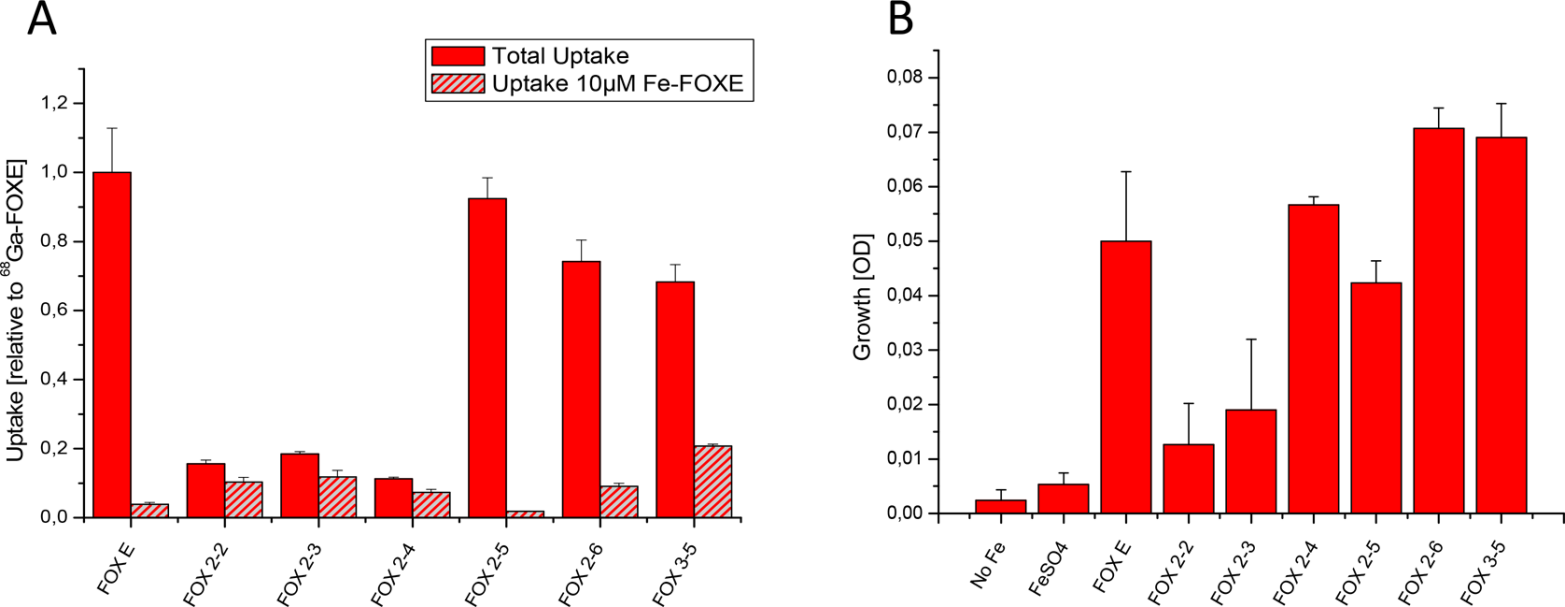
Biological activity of FOXE-analogs in *S. aureus* cultures. (A) Uptake of [^68^Ga]Ga-FOXE derivatives with and without addition of a 10 μM excess of Fe-FOXE to block transporter specific uptake. (B) Growth promotion of Fe-depleted *S. aureus* cultures by addition of 0.2 μM Fe-FOXE-analogs as iron sources including 0.2 μM FeSO_4_ as control. OD values at 12 h of culture are shown; the time course can be found in Figure S9.

Uptake of ^68^Ga-labeled analogs in iron depleted *S. aureus* cultures are shown in Figure 2A. Interestingly, a high variability in uptake values was found. Whereas [^68^Ga]Ga-FOX 2-5, 2-6, and 3-5 showed comparable uptake to [^68^Ga]Ga-FOXE, which could be effectively blocked with a 10 μM excess of Fe-FOXE, the radiolabeled ligands [^68^Ga]Ga-FOX 2-2, 2-3, and 2-4 revealed considerably lower uptake values and only a minor reduction of activity with an excess of Fe-FOXE, indicating poor transport of these analogs. Growth promotion assays confirmed these results with a growth promotion by Fe-FOX 2-5, 2-6, and 3-5 comparable to Fe-FOXE, whereas Fe-FOX 2-2 and 2-3 provided only limited growth promotion which was only slightly better than that of Fe(II) sulfate alone. Only Fe-FOX 2-4 showed a different behavior and provided comparable growth promotion to Fe-FOXE, even though uptake values were low.

It should be kept in mind that uptake assay provides information on the short-term activity of the siderophores related to uptake, whereas the growth promoted a longer-term activity more related to the iron supply leading to the support of growth. Therefore, Fe-FOX 2-4 seems to be a good Fe-source to the support of growth of *S. aureus*, even though transport is certainly less effective in comparison to the natural siderophore FOXE. Growth, expressed as OD values after 12 h of incubation, is shown in Figure 2B, whereas time course can be found in the Supporting Information (Figure S11). Overall, our results indicate that the size of the cage influences the transport efficiency and growth support, with advantages for the larger ligands in the case of *S. aureus*. However, further studies are necessary to explore potential species and strain differences of uptake selectivity and growth promotion.

## Conclusions

Previous studies on an arsenal of modified derivatives of ferrioxamines produced by bacteria in response to cultivation conditions and availability of precursor materials point to the potential of their synthesis as a common chemical answer to various environmental conditions and the presence of other microbes in the surroundings of the primary organism. An analogous approach has been used in our biomimetic design, where we have modified the cyclic FOXE structure to focus our attention on the size of the cycle investigating its importance for thermodynamic stability of Fe(III) and Ga(III) complexes and recognition by *S. aureus*.

Here, a new series of six FOXE analogs is presented, where ligands differ in ring size and with the hydroxamic acids groups positioned retro in relation to natural FOXE. Coordination characteristics, thermodynamic stability, and complex formation equilibria of FOXE analogs with Fe(III) and Ga(III) ions were thoroughly investigated. The stoichiometry of forming complexes was determined by ESI-MS experiments as a 1:1 metal-to-ligand ratio for all six new derivatives. This was further confirmed in potentiometric and UV–vis spectrophotometric titrations. All compounds are excellent Fe(III) and Ga(III) chelators with stability constants and metal affinity within range of natural siderophores and, therefore, are potentially stable enough to keep the metal ions bound under the pH of human serum. To confront metal binding properties of the investigated derivatives with native siderophore FOXE, we have experimentally established speciation and a stability constant of the Ga(III)-FOXE complex and calculated its affinity toward Ga(III) (pGa). The FOX 2-5 analog, which alters from the natural siderophore FOXE only by the position of binding groups (retro), presented the best and most promising properties, almost identical to the native compound.

Growth promotion assays were used to monitor biological potential of the new derivatives. Biological activity in *S. aureus* of analog compounds with larger cavity was retained. Smaller compounds did not promote growth and were not efficiently recognized by investigated bacterial strains. Overall, our derivatives may hold potential as inert and stable carrier agents for radioactive Ga(III) ions for diagnostic medical applications or interesting starting compounds for bioconjugate synthesis of enhanced antibiotics or targeted antimicrobial agents. Further studies will follow to investigate potential species and strain differences of uptake selectivity and growth promotion.

## Experimental Section

### Materials and Methods

All reagents and solvents were purchased from commercial suppliers (Sigma-Aldrich, Chem-Pur, Merck, Fluka Analytical) and used without further purification.

For synthesis, spectral grade solvents were stored over 3 Å molecular sieves for several days. TLC analysis was performed using aluminum-backed plates (200 μm thickness, F-254 indicator) from Merck, and spots were visualized by UV-light and treatment with ninhydrin solution followed by gentle heating. Products were purified by flash chromatography using high-purity grade silica gel (pore size 60 Å, 230–400 mesh particle size) from SiliCycle. Preparative HPLC was performed on a LC-20AP Shimadzu with an ELSD-LTII detector equipped with a Phenomenex Luna C18 250 × 21 mm, 5 μm column eluted with 20 mL/min flow over 20 min of acetonitrile in water. Solvents were removed using a rotary evaporator.

For physicochemical studies, all solutions were prepared in doubly distilled water. Compounds were weighted using a QUINTIX 125D-1CEU Sartorius analytical balance (precision 0.01 mg). pH was adjusted using a Seven Easy pH-meter Mettler Toledo GmbH equipped with a pH combined glass electrode (Mettler Toledo micro pH electrode LE422) with a gel electrolyte calibrated using standardized buffer solutions. The experiments were carried out at 25.0 ± 0.2 °C. Fe(ClO_4_)_3_ and Ga(ClO_4_)_3_ stock solutions were prepared immediately before use from Fe(ClO_4_)_3_·H_2_O and Ga(ClO_4_)_3_·H_2_O (Aldrich, 99.99%, chlorides <0.005%) in 1 × 10^–2^ M HClO_4_ (VWR Chemicals p.a. 70%) and standardized by ICP-OES (iCAP 7400 Duo ICP-OES, Thermo Fisher Scientific) along with spectrophotometric determination for Fe(ClO_4_)_3_, based on the molar extinction coefficient ε = 4160 M^–1^ cm^–1^ at 240 nm.^67^ An HClO_4_ solution was titrated by standardized NaOH (0.1 M Fluka standard solution). The carbonate-free NaOH solution was standardized by titration with potassium hydrogen phthalate (Merck p.a.). The ionic strength was fixed at *I* = 0.1 M with NaClO_4_ (Aldrich ACS reagent, ≥98.0%). We are fully aware that to obtain reliable data at a very acidic pH (<1) the ionic strength of 0.1 M is not sufficient to keep the ionic activity stable. However, because of a decomposition of hydroxamic acids under strongly acidic conditions,^58,59^ and a large error coming from it, the experiments completed below pH 1 (i) were not taken into account during data evaluation or (ii) treated with precaution and precluded from the discussion.

### Spectroscopic Measurements

NMR spectra were recorded on Bruker Avance DRX 500 (^1^H NMR at 500 MHz and ^13^C NMR at 126 MHz) magnetic resonance spectrometers. ^1^H NMR spectra are reported in chemical shifts downfield from TMS using the respective residual solvent peak as internal standard ((CD_3_)_2_SO δ 2.50 ppm). ^1^H NMR spectra are reported as follows: chemical shift (δ, ppm), multiplicity (s = singlet, d = doublet, t = triplet, q = quartet, dd = doublet of doublets, dt = doublet of triplets, dq = doublet of quartets, m = multiplet), coupling constant (*J*) in Hz, and integration. ^13^C NMR spectra are reported in chemical shifts downfield from TMS using the respective residual solvent peak as internal standard ((CD_3_)_2_SO δ 39.5 ppm). LC-MS analysis (electrospray ionization, ESI) was obtained on a Waters Alliance 2695 separation module with a PDA 2996 UV detector and a Waters Micromass ZQ 2000 mass detector equipped with a Kinetex Biphenyl 50 × 2.1 mm, 2.6 μm column eluted with 0.3 mL/min flow of 3–100% gradient (over 6 min) of acetonitrile in water (mobile phases contained an addition of 0.04% of formic acid).

### ESI-MS

Electrospray mass spectrometry (ESI-MS) was performed on a Bruker Apex Ultra FT-ICR and MicrO-TOF-Q mass spectrometer. The instrumental parameters were as follows: scan range, *m*/*z* 200–1600; dry gas, nitrogen; temperature, 170 °C; capillary voltage 4500; ion energy, 5 eV.

The capillary voltage was optimized to the highest signal-to-noise ratio. The spectra were recorded in the positive mode. The compounds were dissolved in the MeOH/H_2_O solution (50/50 by weight); the same solvent mixture was used to dilute the matrix solutions to the concentration range 1 × 10^–5^ M. The stock solutions of Fe(III) and Ga(III) were prepared accordingly to a previously described procedure, and complexes of a 1:1 metal-to-ligand molar ratio were formed by adding them to ligand solutions following by pH adjustment to 3 or 7 by addition of HClO_4_ and NaOH. pH accuracy was provided by a Beckman Φ72 pH-meter equipped with a combined glass electrode and standardized by a classical method with buffers.

### Potentiometric and pH-Dependent UV–Visible Titration

Proposed biomimetic analogs of cyclic siderophore FOXE bind Fe(III) ions starting from pH around 0, and the complexation is essentially complete far below or around pH of 2. Therefore, the stability constants of the first species formed in solution were determined from the spectrophotometric pH-dependent batch titrations carried out in the pH range of around 0.1–2 (Figure S3), by following the changes in the intense LMCT band of the metal complex. These values were further used as constant values in spectrophotometric or potentiometric titrations carried out in the pH range from 2 to 11 (Figure S3) to get the overall stability constants, logβ_FeL_ (Table S5). Since Ga(III)-FOX complexes present similar coordination properties, the first species start to form below pH 2 as well. However, in this case, we have used indirect methods to determine stability constants for Ga(III)-FOXE analogs and the Ga(III) complex with native FOXE, as Ga(III) complexes are spectroscopically silent in the UV–vis region. Metal–metal competition titrations were carried out at pH 1.6 for FOX 2-5 and FOX 2-4 and at pH 2 for the rest of the analogs and the native compound. The intense LMCT band of the Fe(III) complex was silenced after addition of a fixed volume of Ga(III) ions (Figure S5).

### Potentiometric Titrations

Potentiometric titrations were performed using an automatic titrator system Titrando 905 (Metrohm) equipped with a 800 Dosino dosing unit and a microburet with a volume of 2 mL. A combined glass electrode was calibrated each day for hydrogen ion concentration by 0.1 M HClO_4_ with CO_2_-free 0.1 M NaOH solutions. A stream of high purity grade argon, presaturated with water vapor, passed over the surface of the solution cell, filled with 3 mL of the studied solution. For each system, a minimum of four independent titrations was performed. The purity of ligand and accurate concentration of ligand solution were determined before each experiment with the Gran method^68^ by potentiometric titration of the 3 mL sample of the ligand, with [L] ∼ 1 × 10^–3^ M. In separate potentiometric titrations, the metal-to-ligand ratio was 1:1, and the concentration of metal was 2 × 10^–3^ M. The potentiometric data were refined to obtain the overall Fe(III)-FOXE and Ga(III)-FOXE derivative binding constants (logβ_Fe(III)L_, logβ_Ga(III)L_).

### pH-Dependent UV–Vis Titrations

The absorption measurements (200 nm–800 nm) were performed on a Varian Cary 300 Bio UV–visible spectrophotometer. The solutions were measured in high precision Quartz SUPRASIL cuvettes (1 and 3 mL) with 1 cm lightpath (Hellma).

Two series of experiments were performed: (i) in the pH range of 0.1–2 and (ii) between pH 2 and 11. In the first series of experiments, (i) the stock solution of the ligand was divided into various batches followed by Fe(III) addition, with a constant total volume of 2 mL, [Fe(III)] = 2 × 10^–4^ M, and a metal-to-ligand molar ratio of 1:1. The pH was controlled by concentration of the perchloric acid. After preparation, each solution was allowed to equilibrate for 1 h at 25 °C, and then its UV–vis spectrum was recorded. In the second set of experiments (ii) 3 mL of solution containing a 1:1 molar ratio of the Fe(III)-FOXE analog, [Fe(III)] = 2 × 10^–4^ M, was titrated within a pH range of 2–11 by addition of known volumes of 0.1 M NaOH, but data was analyzed only up to pH 9, as above this pH, the hydrolysis of complex occurred, causing a decrease of the intensity of spectra.

### Ligand–Ligand Competition UV–Vis Titrations (Competition Experiments with EDTA)

In this series of experiments, the stock solution of FOX ligand was divided into various batches followed by metal addition with a constant total volume of 2 mL, [Fe(III)] = 2 × 10^–4^, and a metal-to-ligand molar ratio of 1:1. The EDTA ligand was added to a previously prepared series of solutions in increasing concentration, from a 0 to 10 molar excess for FOX 2-5 and from a 0 to 1.2 molar excess for the rest of the derivatives. pH was adjusted to ∼7 with the NaOH solution. After preparation, each solution was allowed to equilibrate for 3–7 days, depending on the investigated complex. Changes were observed between the collected spectra, and they were measured consequently until no further changes were present. The UV–vis spectra were recorded (Figure S4), and competition data were refined to obtain the overall Fe(III)-FOXE derivative binding constant (logβ_Fe(III)L_). The protonation constants of FOXE derivatives (Table S4) and formation constants for Fe(III)-FOXE derivative complex species (Table S5) were treated as fixed parameters in data analysis.

### Metal–Metal Competition UV–Vis Titrations

Metal competition assays of Fe(III)-FOXE and Fe(III)-FOX systems were carried out as a function of Ga(III) concentration in the 250–700 nm range. Fifteen sample solutions with a constant concentration of the Fe(III)-FOXE derivative or Fe(III)-FOXE (1.6 × 10^–4^ M) were titrated by Ga(III) ions (starting from 0 mol equiv up to an 800 molar excess) at constant pH (1.6 or 2). UV–vis spectra of prepared solutions were recorded after equilibrium was reached after at least 1 h. The samples were measured again after 24, 48, and 72 h, and vials were kept in the dark in ambient temperature. Major changes were observed between samples measured after 1 h and 24 h. After this time, collected spectra were not characterized by any analytical changes. Data obtained in competition experiments were refined to determine overall Ga(III)-FOXE derivatives or Ga(III)-FOXE binding constant (logβ_Ga(III)HL_ or logβ_Ga(III)L_, depending on the ligand).

### UV–Vis pH-Metric Titrations on Ligand Bands

A stock solution of the ligand was divided into various batches followed by Ga(III) addition, with a constant total volume of 2 mL, [Fe(III)] = 5 × 10^–5^ M, and a metal-to-ligand molar ratio of 1:1. In the pH range from 0.1 to 2, the pH was controlled by concentration of the HClO_4_. After preparation, each solution was allowed to equilibrate for 1 h at 25 °C, and then its UV–vis spectrum was recorded. In the second set of experiments, 3 mL of solution containing a 1:1 molar ratio of the Ga(III)-FOXE analog, [Fe(III)] = 5 × 10^–5^ M, was titrated directly in a quartz cuvette, in the pH range of 2–11 by addition of known volumes of 0.1 M NaOH. The spectroscopic data (Figure S6) were refined to obtain a p*K* value of the Ga(III)-FOX 2-5 complex.

### Cyclic Voltammetry

Redox potentials for Fe(III)-FOXE derivatives were determined using cyclic voltammetry with a VA 797 Computrace Voltammeter (Metrohm) equipped with glass-carbon, AgCl/Cl, and platinum wire electrodes. Concentration of the measured samples was [Fe(III)] = 2 × 10^–3^, M:L ratio 1:1, at 25.0 ± 0.1 °C; pH 7.0 was provided by addition of a suitable amount of NaOH under control of a pH meter. The scan range was from −0.3 V to −1 V in a cycle: Fe(III)–FOX  Fe(II)–FOX Fe(III)–FOX. Received data (Figure S8) was standardized for the NHE electrode. The electrochemical data were refined to obtain the overall Fe(II)-FOXE derivative binding constant (logβ_Fe(II)L_^–^) by implementing the Nernst equation (S1).

### Analysis and Processing of the Data

The experimental sets with over 140 points collected in the pH range 2–11 for a single potentiometric measurement were imported to the SUPERQUAD software,^69^ which uses nonlinear least-squares methods for data treatment. Spectroscopic data were refined using SPECFIT/32 software, which uses factor analysis to reduce the absorbance matrix and to extract the eigenvalues prior to the multiwavelength fit of the reduced data set according to the Marquardt algorithm and adjusts the absorptivity and the stability constants of the species formed at equilibrium.^70−72^ Standard deviation was used for the calculation of the uncertainties in logβ. Speciation plots were prepared using HYSS software,^73^ implementing data gathered in Tables S4, S5, and S8. Raw data was processed using Origin 7.0. All plots were prepared using Origin 7.0. MS data and calculated isotope patterns were prepared using Bruker Compass Data Analysis 4.0 software.

In the calculations of complex stability constants, the protonation constants of free ligands and the hydrolysis constants related to Fe(OH)^2+^ (logβ_11_ = −2.56), Fe(OH)_2_^+^ (logβ_12_ = −6.20), Fe_2_(OH)_2_^4+^ (logβ_22_ = −2.84), and Fe(OH)_3_(logβ_13_ = −11.41) and to GaOH^2+^ (logβ_11_ = −3.11), Ga(OH)_2_^+^ (logβ_12_ = −7.66), Ga(OH)_3_ (logβ_13_ = −11.07), and Ga(OH)_4_^–^ (logβ_14_ = −15.66) species were taken into account.^74^ The experimental wavelength window was beyond the range of absorption of the hydrolytic forms of Fe(III) ion which are characterized by λ max below 300 nm. Nevertheless, we have included the spectrum of Fe(III) in water solvent at the investigated pH range as fixed in the calculation model.

### Radiolabeling

Gallium-68 was produced by fractionated elution of a ^68^Ge/^68^Ga generator (IGG100, Eckert & Ziegler Isotope Products, Berlin, Germany; nominal activity of 1850 MBq) with 0.1 M hydrochloric acid (HCL, Rotem Industries, Arva, Israel). For labeling, 10 μg (5–8 nmol) of FOXE derivative was mixed with 200 μL of gallium eluate (∼15–30 MBq), and the pH was adjusted to 4.5 by adding 20 μL of sodium acetate solution (1.14 M) per 100 μL of eluate. The mixture was left to react for 10 min at RT and finally analyzed by radio TLC and radio RP HPLC.^20^

RP-HPLC analysis was performed with the following instrumentation: UltiMate 3000 RS UHPLC pump, UltiMate 3000 autosampler, Ultimate 3000 column compartment (25 °C oven temperature), UltiMate 3000 variable wavelength detector (Dionex, Germering, Germany; UV detection at λ = 220 nm), GABI Star radiometric detector (Raytest GmbH, Straubenhardt, Germany), Jupiter 5 μm C18 300 Å 150 × 4.6 mm (Phenomenex Ltd., Aschaffenburg, Germany) column with acetonitrile (ACN)/H_2_O/0.1% trifluoroacetic acid (TFA) as mobile phase; flow rate of 1 mL/min; gradient 0.0–3.0 min 0% ACN, 3.0–14 min 0–50% ACN, 14.0–16.0 min 50% ACN. Radiolabeling efficiency and RCP were additionally analyzed by ITLC-SG (Varian, Lake Forest, CA, USA) with a 0.1 M citrate buffer pH 5 as mobile phase. The ITLC-SG strips were scanned using a RadioTLC-Scanner (Scanram, Lablogic, Sheffield, UK).

In the case of radiolabeling yields below 95%, samples were purified by solid phase extraction (SPE). Sep-Pak columns (tC18 Plus Light Cartridge, Waters, Austria) were activated with 1 mL of ethanol and washed with 2 mL of water. Radiolabeled samples were applied, and unbound ^68^Ga was removed by washing with 2 mL of water. ^68^Ga-labeled compounds were eluted with 0.25 mL of 70% ethanol and diluted with water for further investigations. SPE results were also used to calculate RCP as percentage of bound (eluted in the ethanol fraction) over total activity eluted.

### Distribution Coefficient (LogD)

For the determination of the lipophilicity of the gallium complexes, we have delivered the distribution coefficient between octanol and the PBS buffer of ^68^Ga-labeled ligands. After dissolution of the investigated radiolabeled complexes in PBS to a concentration of approximately 9 μM, 50 μL of this solution was added to 450 μL of PBS and 500 μL of octanol into an Eppendorf tube followed by vigorous shaking of the two phases for 20 min at 1400 rpm at room temperature (MS 3 basic vortexer, IKA, Staufen, Germany) followed by centrifugation for 2 min at 4500 rpm (Eppendorf Centrifuge 5424, Eppendorf AG, Hamburg, Germany). After that, 200 μL of each phase was collected and measured in a 2480 automatic Gamma counter Wizard 2 3″ (PerkinElmer, Waltham, MA, USA). LogD values were calculated by dividing measured values of octanol by water and logarithmizing the result. Values > 0 reflect lipophilic compounds; values < 0 reflect hydrophilic compounds (*n* = 3, six technical replicates).

### Uptake Assay in *S. aureus*

*S. aureus* was cultured in RPMI1640 medium at 37 °C for 40 h without additional iron to generate iron starvation. For uptake assays, 180 μL of *S. aureus* culture was added to prewetted 96-well MultiScreen Filter Plates HTS (1 μm glass fiber filter, Merck Millipore, Darmstadt, Germany) and incubated for 15 min at 37 °C with either 25 μL of PBS or 25 μL of [Fe]FOXE blocking solution (∼10 μM) as control. After that, 50 μL of investigated radiolabeled ligands of final concentration about 90 nM was added and left for incubation for another 45 min at 37 °C. Filters were washed twice with ice cold TRIS buffer followed by a measurement in the gamma counter (Wizard 2 3″, PerkinElmer, Waltham, MA, USA). All derivatives were tested in quadruplicate.

### Growth Promotion Assay in *S. aureus*

*S. aureus* was grown in RPMI1640 with dipyridyl (final concentration 200 μM) and the Fe-siderophore of interest (final concentration 0.2 μM) at 37 °C. Optical density (OD) was measured at 600 nm to monitor bacterial growth in the log-phase (after 11 h). All derivatives were tested in triplicate.

## Supporting Information Available

Detailed data including synthetic procedures, NMR spectra of investigated compounds, ESI-MS spectra, cyclic voltammetry (CV) and UV–vis spectroscopic data from solution studies, details of Fe(III) and Ga(III) complex solution studies, sample chromatograms of HPLC and TLC for radiolabeling studies, and complementary growth promotion assay data (PDF)
