# Supplementary material for: Cyclic Analogs of Desferrioxamine E Siderophore for 68Ga Nuclear Imaging: Coordination Chemistry and Biological Activity in Staphylococcus aureus
Source: Inorg Chem. 2021 Nov 16;60(23):17846–57. doi: 10.1021/acs.inorgchem.1c02453 (PMC8653149; doi:10.1021/acs.inorgchem.1c02453)
Supplement: Supplementary file 1 — ic1c02453_si_001.pdf [file ic1c02453_si_001.pdf]

Supporting Information

Cyclic analogs of desferrioxamine E siderophore for ^{68}Ga nuclear imaging: coordination chemistry and biological activity in *Staphylococcus aureus*

Andrzej Mular,[a] Abraham Shanzer,[b] Henryk Kozłowski,[a,c] Isabella Hubmann,[d] Matthias Misslinger,[e] Julia Krzywik,[f] Clemens Decristoforo,[d] and Elzbieta Gumienna-Kontecka [a*]

[a] Faculty of Chemistry, University of Wrocław, 50-383 Wrocław, Poland

Email: elzbieta.gumiennakontecka@chem.uni.wroc.pl, orcid.org/0000-0002-9556-6378

[b] Department of Organic Chemistry, The Weizmann Institute of Science, Rehovot 7610001, Israel

[c] Department of Health Sciences, University of Opole, Katowicka 68, 45-060 Opole, Poland

[d] Department of Nuclear Medicine, Medical University Innsbruck, A-6020 Innsbruck, Austria

[e] Institute of Molecular Biology, Medical University Innsbruck, A-6020 Innsbruck, Austria

[f] TriMen Chemicals, Piłsudskiego 141, 92-318 Łódź, Poland

Synthetic procedures and characterization data for the prepared compounds

The general route for the synthesis of intermediates **1** and **2** is depicted in Scheme S1.

analog	x =	y =
FOX 2-2	2	2
FOX 2-3	2	3
FOX 2-4	2	4
FOX 2-5	2	5
FOX 2-6	2	6
FOX 3-5	3	5

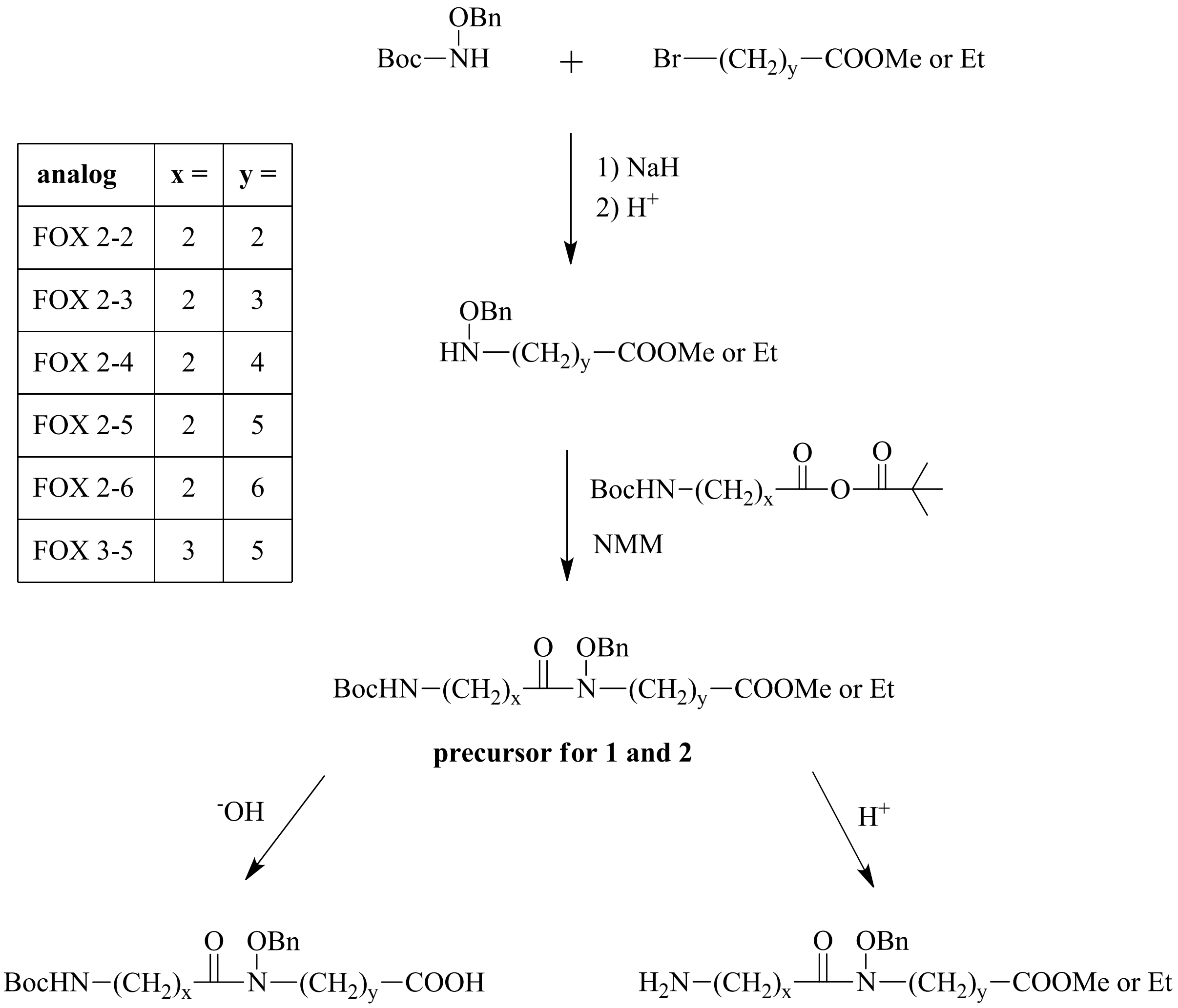

1 **2**

Scheme S1. The general route for the synthesis of **1** and **2**.

General procedure for the synthesis of precursor for compounds 1 and 2

To a solution of *tert*-butyl benzyloxycarbamate (1.0 equiv.) in DMF (5 ml / 1 mmol), 50% NaH (1.1 equiv.) was added portionwise, and the reaction was stirred at RT for 30 min. After 30 min, appropriate bromoester (1.1 equiv.) was added. Reaction was heated at 70°C and its progress was monitored by LC-MS. Then poured into mixture of ice and water and the product was extracted three times by hexane. The hexane extracts were washed with brine, dried over

$MgSO_4$ and concentrated *in vacuo* to give the respective product as yellowish oil with a yield of 80-90%, depending on bromoester used.

Next, the oil was diluted with small amount of dioxane and 3M HCl/dioxane (10 ml / 1 g of compound) was added. Reaction progress was monitored by LC-MS. Then the reaction mixture was concentrated under reduced pressure and evaporated thrice from hexane to give the amine in a quantitative yield.

To pivaloylic anhydride of Boc-β-alanine or Boc-γ-aminobutyric acid (1.0 equiv.) in THF (tetrahydrofuran) (5 ml / 1 mmol), the solution of amine obtained above (1.0 equiv.) in THF was added along with NMM (*N*-methylmorpholine, 3.0 eq). Reaction was stirred at RT and its progress was monitored by LC-MS. Then the reaction mixture was diluted with EtOAc, washed twice with H_2O, twice with 2M HCl, twice with 5% $NaHCO_3$, brine and dried over $MgSO_4$. The residue was purified using column flash chromatography (silica gel; EtOAc/hexanes) to give the pure precursor for **1** and **2** as an oil.

Results

Precursor with **x = 2** and **y = 2**, methyl ester: Yield: 47%; LC-MS t_R = 4.5 min, ESI-MS (*m/z*): $[M+Na]^+$ = 403, $[M\text{-}Boc]^+$ = 281

Precursor with **x = 2** and **y = 3**, ethyl ester: Yield: 58%; LC-MS t_R = 4.7 min, ESI-MS (*m/z*): $[M+Na]^+$ = 431, $[M\text{-}Boc]^+$ = 309

Precursor with **x = 2** and **y = 4**, methyl ester: Yield: 79%; LC-MS t_R = 4.8 min, ESI-MS (*m/z*): $[M+Na]^+$ = 431, $[M\text{-}Boc]^+$ = 309

Precursor with **x = 2** and **y = 5**, methyl ester: Yield: 57%; LC-MS t_R = 4.8 min, ESI-MS (*m/z*): $[M+Na]^+$ = 445, $[M\text{-}Boc]^+$ = 323

Precursor with **x = 2** and **y = 6**, ethyl ester: Yield: 78%; LC-MS t_R = 5.2 min, ESI-MS (*m/z*): $[M+Na]^+$ = 473, $[M\text{-}Boc]^+$ = 351

Precursor with **x = 3** and **y = 5**, ethyl ester: Yield: 76%; LC-MS t_R = 5.1 min, ESI-MS (*m/z*): $[M+Na]^+$ = 473, $[M\text{-}Boc]^+$ = 351

General procedure for the synthesis of compound 1

To a solution of precursor for 1 and 2 (1.0 equiv.) in MeOH (5 ml / 1 mmol), 1M NaOH (2.0 equiv.) was added and the reaction was stirred at RT. Reaction progress was monitored by LC-MS. Then the reaction mixture was diluted with H_2O, MeOH was evaporated and the residues

was acidified with 2M HCl to pH~1-2 and extracted twice with EtOAc. The organic layers were combined, washed with brine, dried over $MgSO_4$ and concentrated *in vacuo* to give the respective product.

Results:

Compound **1** with **x = 2** and **y = 2**: Yield: quantitative; LC-MS t_R = 4.0 min, ESI-MS (*m/z*): $[M+Na]^+$ = 389, $[M\text{-}Boc]^+$ = 267, $[M\text{-}H]^-$ = 365

Compound **1** with **x = 2** and **y = 3**: Yield: quantitative; LC-MS t_R = 4.1 min, ESI-MS (*m/z*): $[M+Na]^+$ = 403, $[M\text{-}H]^-$ = 379

Compound **1** with **x = 2** and **y = 4**: Yield: quantitative; LC-MS t_R = 4.2 min, ESI-MS (*m/z*): $[M+Na]^+$ = 417, $[M\text{-}H]^-$ = 393

Compound **1** with **x = 2** and **y = 5**: Yield: quantitative; LC-MS t_R = 4.3 min, ESI-MS (*m/z*): $[M+Na]^+$ = 431, $[M\text{-}Boc]^+$ = 309, $[M\text{-}H]^-$ = 407

Compound **1** with **x = 2** and **y = 6**: Yield: quantitative; LC-MS t_R = 4.5 min, ESI-MS (*m/z*): $[M+Na]^+$ = 445, $[M\text{-}Boc]^+$ = 323, $[M\text{-}H]^-$ = 421

Compound **1** with **x = 3** and **y = 5**: Yield: quantitative; LC-MS t_R = 4.5 min, ESI-MS (*m/z*): $[M+Na]^+$ = 445, $[M\text{-}H]^-$ = 421

General procedure for the synthesis of compound 2

The precursor for **1** and **2** was diluted with small amount of dioxane and 3M HCl/dioxane (10 ml / 1 g of compound) was added. Reaction progress was monitored by LC-MS. Then the reaction mixture was concentrated under reduced pressure and evaporated thrice from hexane to give the respective product.

Results:

Compound **2** with **x = 2** and **y = 2**, methyl ester

Yield: quantitative

LC-MS t_R = 2.5 min, ESI-MS (*m/z*): $[M+H]^+$ = 281, $[M+Na]^+$ = 303

Compound **2** with **x = 2** and **y = 3**, ethyl ester

Yield: quantitative

LC-MS t_R = 2.8 min, ESI-MS (*m/z*): $[M\text{-}H]^-$ = 309

Compound **2** with **x = 2** and **y = 4**, methyl ester

Yield: quantitative

LC-MS t_R = 2.8 min, ESI-MS (*m/z*): $[M+H]^+$ = 309

Compound **2** with **x = 2** and **y = 5**, methyl ester

Yield: quantitative

LC-MS t_R = 3.1 min, ESI-MS (*m/z*): $[M+H]^+$ = 323

Compound **2** with **x = 2** and **y = 6**, ethyl ester

Yield: quantitative

LC-MS t_R = 3.3 min, ESI-MS (*m/z*): $[M+H]^+$ = 351, $[M+Na]^+$ = 373

Compound **2** with **x = 3** and **y = 5**, ethyl ester

Yield: quantitative

LC-MS t_R = 3.3 min, ESI-MS (*m/z*): $[M+H]^+$ = 351, $[M+Na]^+$ = 373

General procedure for the synthesis of compound 3

To a solution of compound **1** (1.0 equiv.) in DCM (5 ml / 1 mmol), TBTU (1.0 equiv.), TEA (3.0 equiv.) and compound **2** (1.0 equiv.) were added. Reaction was stirred at RT and its progress was monitored by LC-MS. Then the reaction mixture was diluted with EtOAc, washed twice with H_2O, twice with 2M HCl, twice with 5% $NaHCO_3$, brine dried over $MgSO_4$ and concentrated *in vacuo* to give the respective product. Next, the compound was diluted with small amount of dioxane and 3M HCl/dioxane (10 ml / 1 g of compound) was added. Reaction progress was monitored by LC-MS. Then the reaction mixture was concentrated under reduced pressure and evaporated thrice from hexane to give the respective product.

Results:

Compound **3** with **x = 2** and **y = 2**, methyl ester

Yield: 89%

LC-MS t_R = 3.3 min, ESI-MS (*m/z*): $[M+H]^+$ = 529, $[M+HCOO]^-$ = 573

Compound **3** with **x = 2** and **y = 3**, ethyl ester

Yield: 84%

LC-MS t_R = 3.7 min, ESI-MS (*m/z*): $[M+H]^+$ = 571, $[M-HCOO]^-$ = 615

Compound **3** with **x = 2** and **y = 4**, methyl ester

Yield: 92%

LC-MS t_R = 3.6 min, ESI-MS (*m/z*): $[M+H]^+$ = 585, $[M-Na]^+$ = 607, $[M-HCOO]^-$ = 629

Compound **3** with **x = 2** and **y = 5**, methyl ester

Yield: 93%

LC-MS t_R = 3.7 min, ESI-MS (*m/z*): $[M+H]^+$ = 613, $[M-Na]^+$ = 635, $[M+HCOO]^-$ = 657

Compound **3** with **x = 2** and **y = 6**, ethyl ester

Yield: 85%

LC-MS t_R = 3.1 min, ESI-MS (*m/z*): $[M+H]^+$ = 655

Compound **3** with **x = 3** and **y = 5**, ethyl ester

Yield: 87%

LC-MS t_R = 4.1 min, ESI-MS (*m/z*): $[M+H]^+$ = 655, $[M+HCOO]^-$ = 699

General procedure for the synthesis of compound 4

To a solution of compound **1** (1.0 equiv.) in DCM (5 ml / 1 mmol), TBTU (1.0 equiv.), TEA (3.0 equiv.) and compound **3** (1.0 equiv.) were added. Reaction was stirred at RT and its progress was monitored by LC-MS. Then the reaction mixture was diluted with EtOAc, washed twice with H_2O, twice with 2M HCl, twice with 5% $NaHCO_3$, brine dried over $MgSO_4$ and concentrated *in vacuo*. The residue was purified using column flash chromatography (silica gel; EtOAc/MeOH) to give the pure compound.

To a solution of the above compound (1.0 equiv.) in MeOH (5 ml / 1 mmol), 1M NaOH (2.0 equiv.) was added and the reaction was stirred at RT. Reaction progress was monitored by LC-MS. Then the reaction mixture was diluted with H_2O, MeOH was evaporated and the residues was acidified with 2M HCl to pH~1-2 and extracted twice with EtOAc. The organic layers were combined, washed with brine, dried over $MgSO_4$ and concentrated *in vacuo* to give the respective acid.

The above acid was diluted with small amount of dioxane and 3M HCl/dioxane (10 ml / 1 g of compound) was added. Reaction progress was monitored by LC-MS. Then the reaction mixture was concentrated under reduced pressure and evaporated thrice from hexane to give the respective product.

Results:

Compound **4** with **x = 2** and **y = 2**: Yield: 64%; LC-MS t_R = 3.6 min, ESI-MS (*m/z*): $[M+H]^+$ = 763, $[M-H]^-$ = 761

Compound **4** with **x = 2** and **y = 3**: Yield: 56%, LC-MS t_R = 3.6 min, ESI-MS (*m/z*): $[M+H]^+$ =806, $[M-H]^-$ = 804

Compound **4** with **x = 2** and **y = 4**: Yield: 62%, LC-MS t_R = 3.9 min, ESI-MS (*m/z*): $[M+H]^+$ = 847, $[M-H]^-$ = 845

Compound **4** with **x = 2** and **y = 5**: Yield: 55%; LC-MS t_R = 3.9 min, ESI-MS (*m/z*): $[M+H]^+$ = 889, $[M-H]^-$ = 887

Compound **4** with **x = 2** and **y = 6**: Yield: 62%; LC-MS t_R = 4.1 min, ESI-MS (*m/z*): $[M+H]^+$ = 932, $[M-H]^-$ = 930

Compound **4** with **x = 3** and **y = 5**: Yield: 55%; LC-MS t_R = 4.2 min, ESI-MS (*m/z*): $[M+H]^+$ = 932, $[M-H]^-$ = 930

General procedure for the synthesis of compound 5

To a solution of compound **4** (1.0 equiv.) in DCM (5 dm^3 / 1 mmol), EDCI (1.2 equiv.) and TEA (4.0 equiv.) were added. Reaction was stirred at RT and its progress was monitored by LC-MS. Then the reaction mixture was evaporated and the residue was diluted with EtOAc, washed twice with H_2O, twice with 2M HCl, twice with 5% $NaHCO_3$, brine, dried over $MgSO_4$. Analogs with benzyl groups being the precursors of the final compounds FOX 2-2, FOX 2-3 and FOX 2-4 were directly used in the next transformation. In turn, the precursors of the analogs FOX 2-5, FOX 2-6 and FOX 3-5 were purified using preparative HPLC (MeCN/H_2O).

Results: compound **5** with benzyl groups:

Compound with **x = 2** and **y = 2**: Yield: 47%; LC-MS t_R = 4.1 min, ESI-MS (*m/z*): $[M+H]^+$ = 745, $[M+Na]^+$ = 767, $[M-H]^-$ = 743, $[M+HCOO]^-$ = 789

Compound **4** with **x = 2** and **y = 3**: Yield: 45%; LC-MS t_R = 4.3 min, ESI-MS (*m/z*): $[M+H]^+$ = 787, $[M+Na]^+$ = 810, $[M+HCOO]^-$ = 831

Compound with **x = 2** and **y = 4**: Yield: 55%; LC-MS t_R = 4.5 min, ESI-MS (*m/z*): $[M+H]^+$ = 830, $[M+Na]^+$ = 852, $[M+HCOO]^-$ = 873

Compound with **x = 2** and **y = 5**: Yield: 44%; LC-MS t_R = 4.8 min, ESI-MS (*m/z*): $[M+H]^+$ = 872, $[M+HCOO]^-$ = 916

Compound with **x = 2** and **y = 6**: Yield: 13%; LC-MS t_R = 4.9 min, ESI-MS (*m/z*): $[M+H]^+$ = 914, $[M+HCOO]^-$ = 958

Compound with **x = 3** and **y = 5**: Yield: 22%; LC-MS t_R = 5.0 min, ESI-MS (*m/z*): $[M+H]^+$ = 914, $[M+HCOO]^-$ = 958

10% Pd on carbon catalyst was added to a solution of above compound in dioxane/H_2O (4/1 *v/v*) and the reaction was stirred under 1 atmosphere of H_2 (its progress was monitored by LC-MS), followed by filtration of the catalyst and evaporation of the solvent. Lyophilization from dioxane gave the pure product **5**.

Results: compound **5**:

Compound **5** with **x = 2** and **y = 2**: Yield: 82%; LC-MS t_R = 1.0 min, ESI-MS (*m/z*): $[M+H]^+$ = 475, $[M-H]^-$ = 473

Compound **5** with **x = 2** and **y = 3**: Yield: 88%; LC-MS t_R = 1.7 min, ESI-MS (*m/z*): $[M+H]^+$ =517, $[M-H]^-$ = 515

Compound **5** with **x = 2** and **y = 4**: Yield: 83%; LC-MS t_R = 2.0 min, ESI-MS (*m/z*): $[M+H]^+$ = 559, $[M-H]^-$ = 557

Compound **5** with **x = 2** and **y = 5**: Yield: 81%; LC-MS t_R = 2.4 min, ESI-MS (*m/z*): $[M+H]^+$ = 601, $[M-H]^-$ = 599

Compound **5** with **x = 2** and **y = 6**: Yield: 90%; LC-MS t_R = 2.7 min, ESI-MS (*m/z*): $[M+H]^+$ = 643, $[M-H]^-$ = 641

Compound **45** with **x = 3** and **y = 5**: Yield: 70%; LC-MS t_R = 2.8 min, ESI-MS (*m/z*): $[M+H]^+$ = 643, $[M-H]^-$ = 641

a)

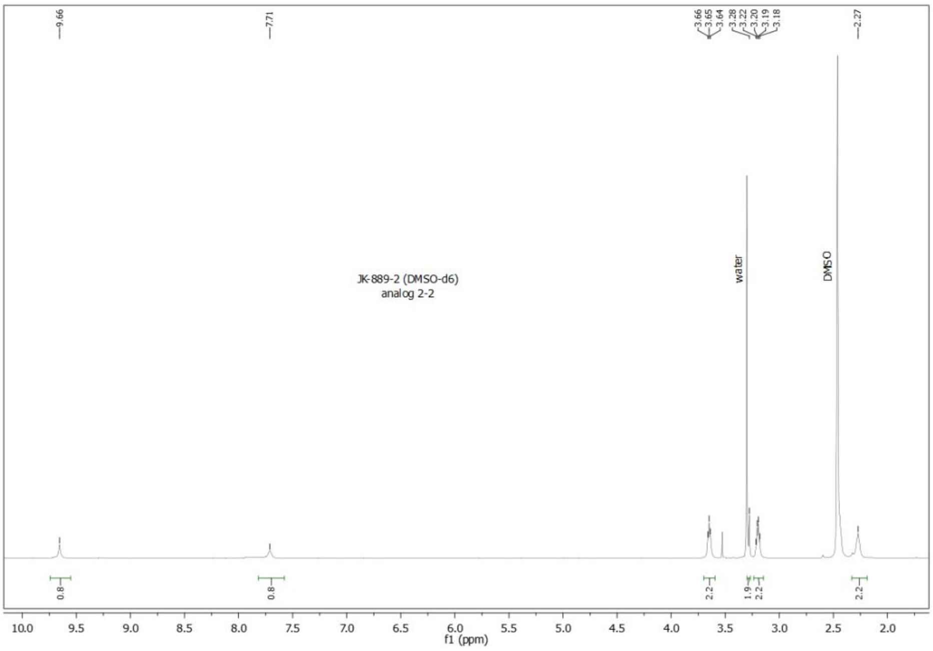

9.66
7.71
3.66
3.65
3.64
3.28
3.22
3.20
3.19
3.18
2.27
JK-889-2 (DMSO-d6)
analog 2-2
water
DMSO
f1 (ppm)

b)

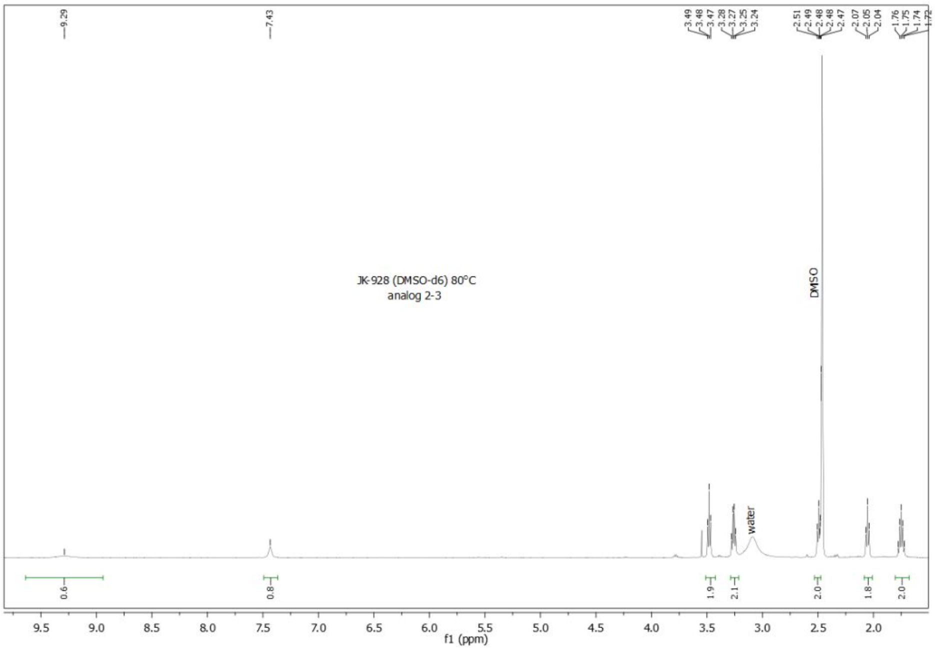

9.29
7.43
3.49
3.48
3.47
3.28
3.27
3.25
3.24
2.51
2.49
2.48
2.48
2.47
2.07
2.05
2.04
1.76
1.75
1.74
1.72
JK-928 (DMSO-d6) 80°C
analog 2-3
DMSO
water
f1 (ppm)

c)

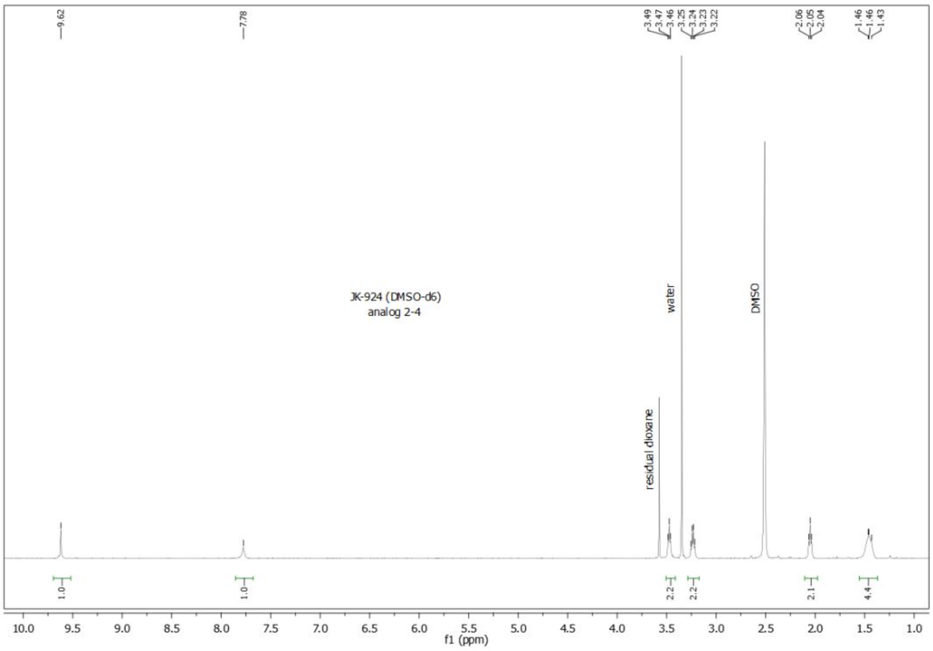
9.62
7.78
3.49
3.47
3.46
3.25
3.24
3.23
3.22
2.06
2.05
2.04
1.46
1.46
1.43
JK-924 (DMSO-d6)
analog 2-4
water
DMSO
residual dioxane
1.0
1.0
2.2
2.2
2.1
4.4
10.0 9.5 9.0 8.5 8.0 7.5 7.0 6.5 6.0 5.5 5.0 4.5 4.0 3.5 3.0 2.5 2.0 1.5 1.0
f1 (ppm)

d)

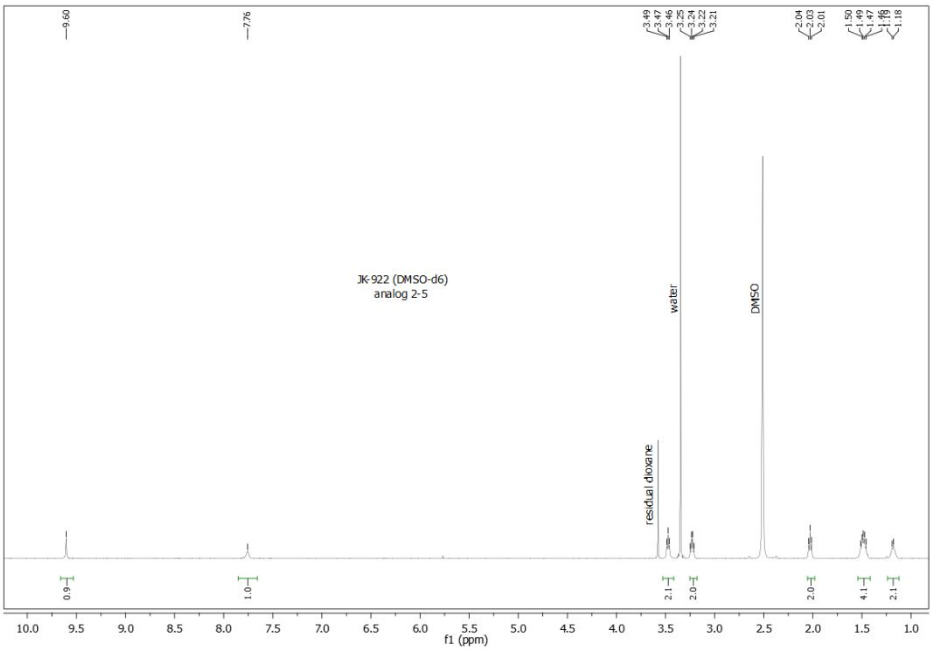
9.60
7.76
3.49
3.47
3.46
3.25
3.24
3.22
3.21
2.04
2.03
2.01
1.50
1.49
1.47
1.18
JK-922 (DMSO-d6)
analog 2-5
water
DMSO
residual dioxane
0.9
1.0
2.1
2.0
2.0
4.1
2.1
10.0 9.5 9.0 8.5 8.0 7.5 7.0 6.5 6.0 5.5 5.0 4.5 4.0 3.5 3.0 2.5 2.0 1.5 1.0
f1 (ppm)

e)

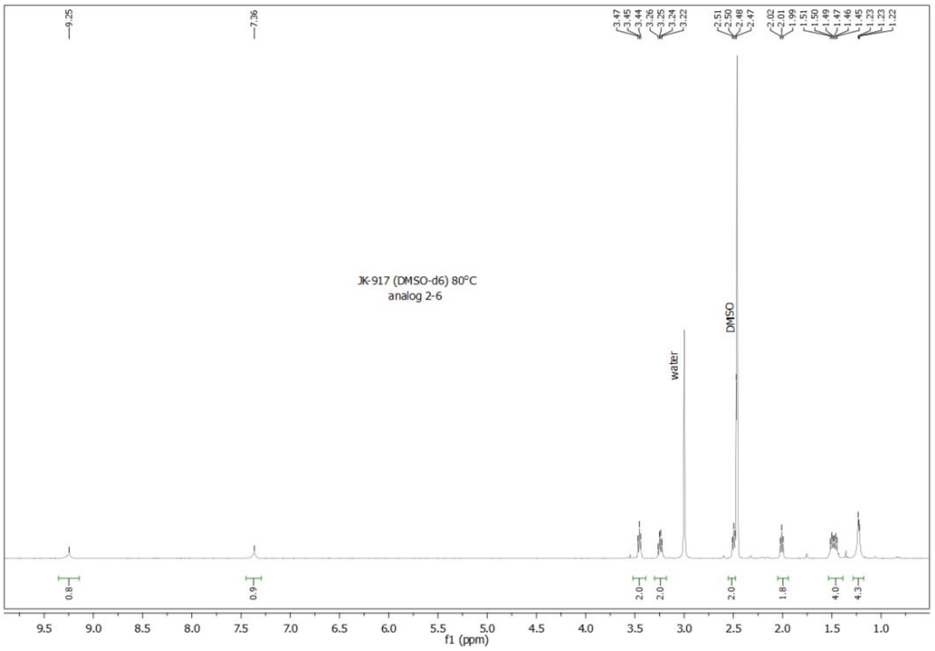

f)

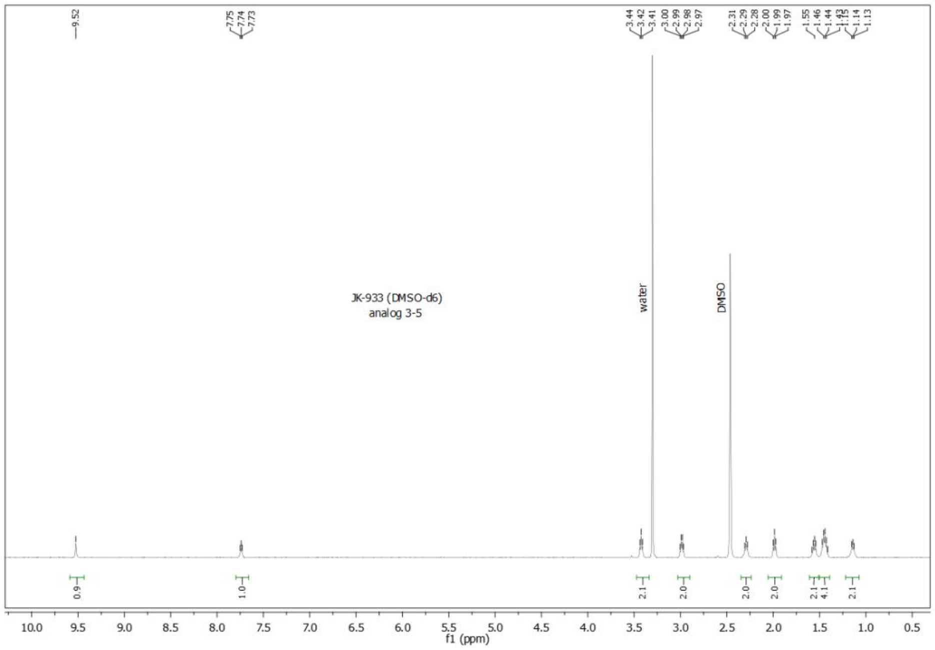

Figure S1. NMR spectra for studied ligands: FOX 2-2(a), FOX 2-3(b), FOX 2-4(c), FOX 2-5(d), FOX 2-6(e) and FOX 3-5(f).

Table S1. Intensity maxima of the major FOX ligands and adduct ions observed by ESI-MS.

Ligand	Pseudomolcular ion	m/z experimental	m/z simulated
FOX 2-2	$\{[H_3L]+[H]^+\}^+$	475.22	475.21
	$\{[H_3L]+[Na]^+\}^+$	497.19	497.19
	$\{[H_3L]+[K]^+\}^+$	513.17	513.17
FOX 2-3	$\{[H_3L]+[H]^+\}^+$	517.26	517.26
	$\{[H_3L]+[Na]^+\}^+$	539.24	539.24
	$\{[H_3L]+[KClO_4]+[H]^+\}^+$	655.16	655.17
FOX 2-4	$\{[H_3L]+[H]^+\}^+$	559.31	559.31
	$\{[H_3L]+[H]^++[KClO_4]\}^+$	697.20	697.22
FOX 2-5	$\{[H_3L]+[H]^+\}^+$	601.35	601.36
	$\{[H_3L]+[Na]^+\}^+$	623.33	623.33
	$\{[H_3L]+[KClO_4]+[H]^+\}^+$	739.25	738.26
FOX 3-5	$\{[H_3L]+[H]^+\}^+$	643.41	643.40
	$\{[H_3L]+[Na]^+\}^+$	665.40	665.39
	$\{[H_3L]+[KClO_4]+[H]^+\}^+$	781.33	781.33

Conditions: [L] = $1 \cdot 10^{-5}$ M, MeOH/H_2O: 50/50 w/w.

All peak assignments were based on the comparison between the calculated and experimental isotope patterns.

Thermodynamic solution studies

ESI-MS measurements

The ESI-MS spectra of the reaction mixture of $Fe(ClO_4)_3$/ligand and $Ga(ClO_4)_3$/ligand with a 1:1 molar ratio (Fig. 2) are characterized by the presence of a few major peaks successfully attributed to the mononuclear species (Table S2, Table S3). Experiments were performed in methanol-water system (50:50 v/v) for better solvent evaporation.

a)

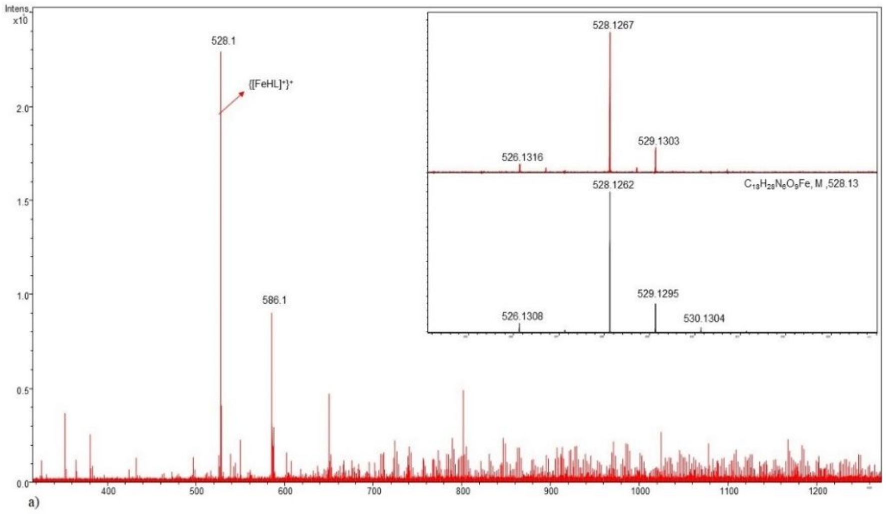

Intens.
x10
528.1
{[FeHL]+}+
586.1
528.1267
529.1303
526.1316
528.1262
C18H28N5O9Fe, M ,528.13
529.1295
526.1308
530.1304
a)

b)

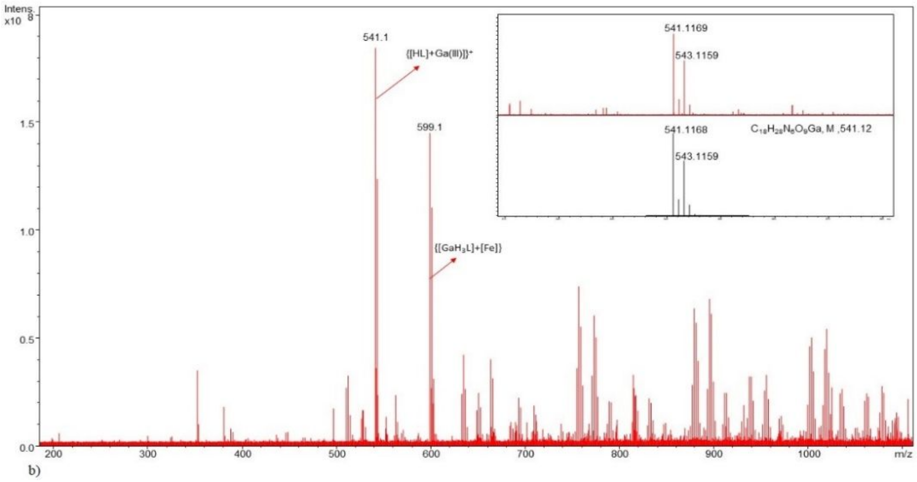

Intens.
x10 8
541.1
{[HL]+Ga(III)]}+
599.1
{[GaH3L]+[Fe]}
541.1169
543.1159
541.1168
C18H28N5O9Ga, M ,541.12
543.1159
m/z
b)

c)

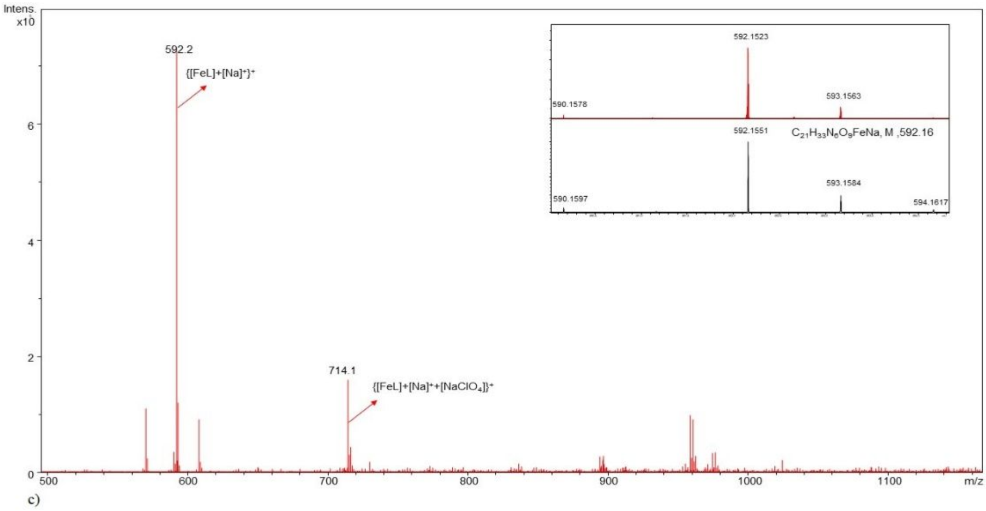

Intens.
x10
592.2
{[FeL]+[Na]+}+
714.1
{[FeL]+[Na]++[NaClO4]}+
592.1523
590.1578
593.1563
592.1551
C21H33N5O9FeNa, M ,592.16
590.1597
593.1584
594.1617
m/z
c)

d)

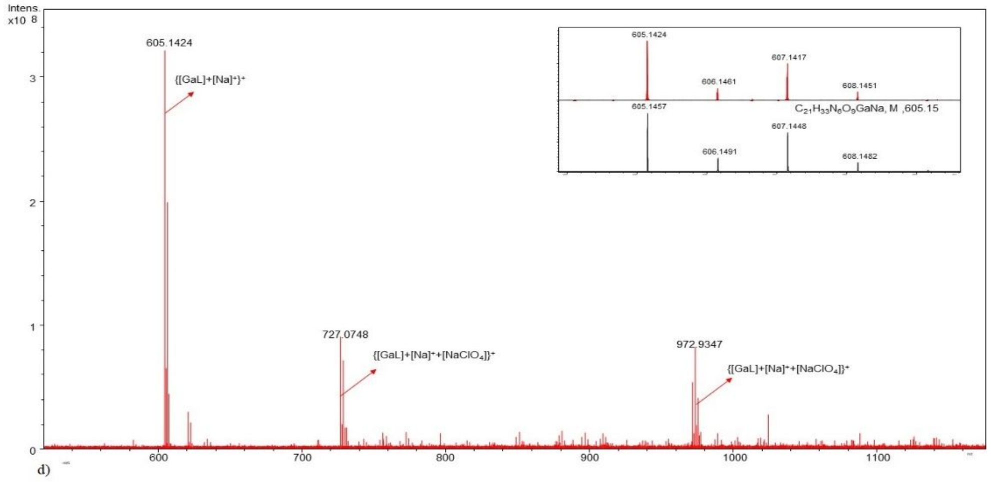
Intens.
x10 8
605.1424
{[GaL]+[Na]+)+
727.0748
{[GaL]+[Na]++[NaClO4]}+
972.9347
{[GaL]+[Na]++[NaClO4]}+
605.1424
606.1461
607.1417
608.1451
605.1457
C21H33N6O9GaNa, M ,605.15
606.1491
607.1448
608.1482
d)

e)

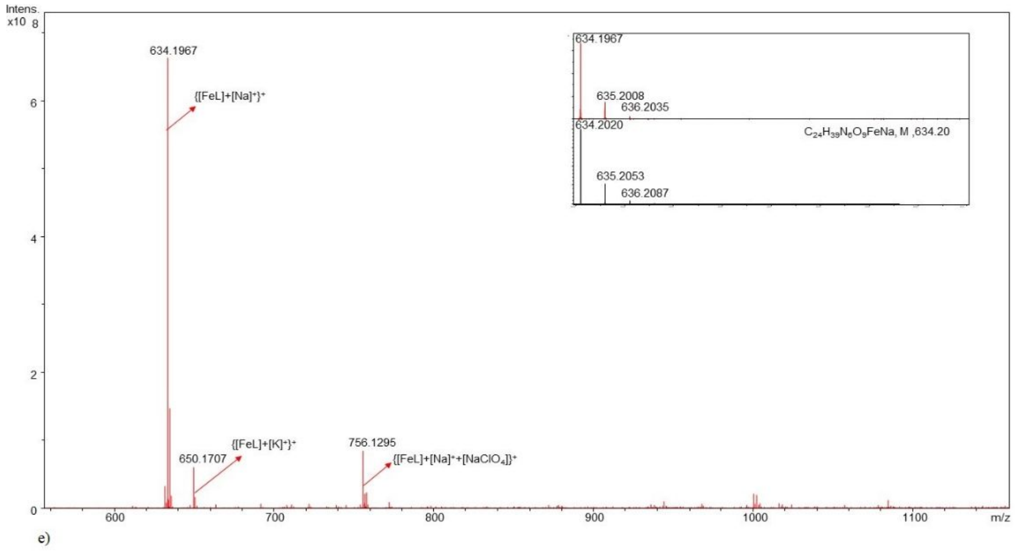
Intens.
x10 8
634.1967
{[FeL]+[Na]+)+
{[FeL]+[K]+)+
650.1707
756.1295
{[FeL]+[Na]++[NaClO4]}+
634.1967
635.2008
636.2035
634.2020
C24H39N6O9FeNa, M ,634.20
635.2053
636.2087
m/z
e)

f)

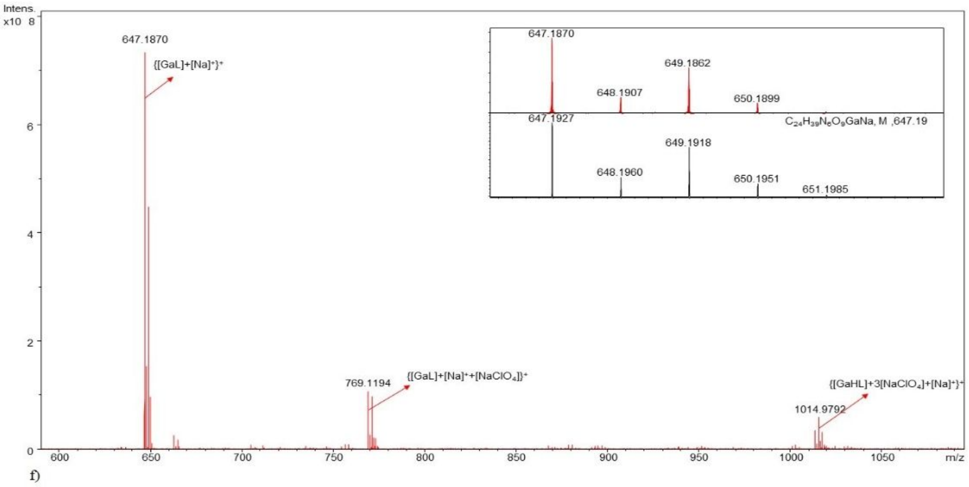
Intens. x10 8
647.1870
{[GaL]+[Na]+}+
769.1194
{[GaL]+[Na]++[NaClO4]}+
{[GaHL]+3[NaClO4]+[Na]+}+
1014.9792
647.1870
649.1862
648.1907
650.1899
647.1927
C24H39N6O9GaNa, M ,647.19
649.1918
648.1960
650.1951
651.1985
m/z
f)

g)

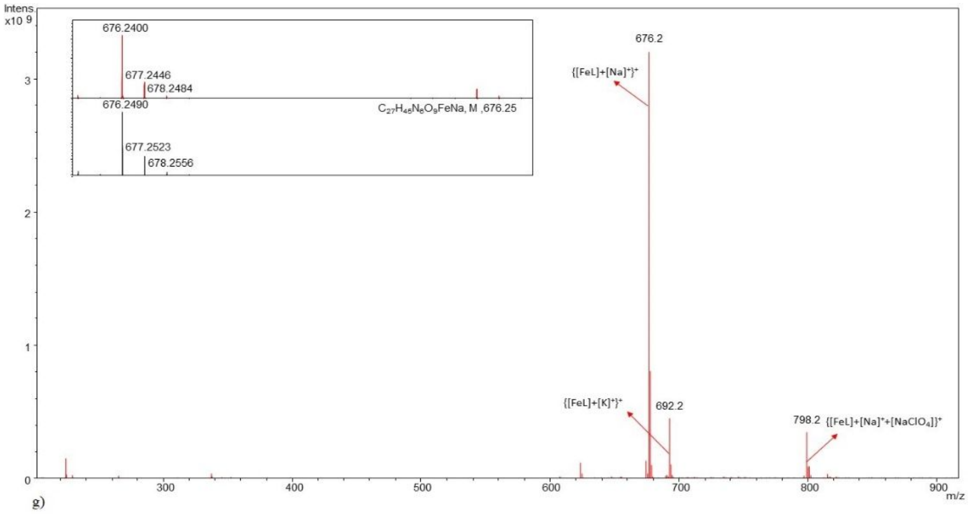
Intens. x10 9
676.2400
677.2446
678.2484
676.2490
C27H45N6O9FeNa, M ,676.25
677.2523
678.2556
676.2
{[FeL]+[Na]+}+
{[FeL]+[K]+}+
692.2
798.2
{[FeL]+[Na]++[NaClO4]}+
m/z
g)

h)

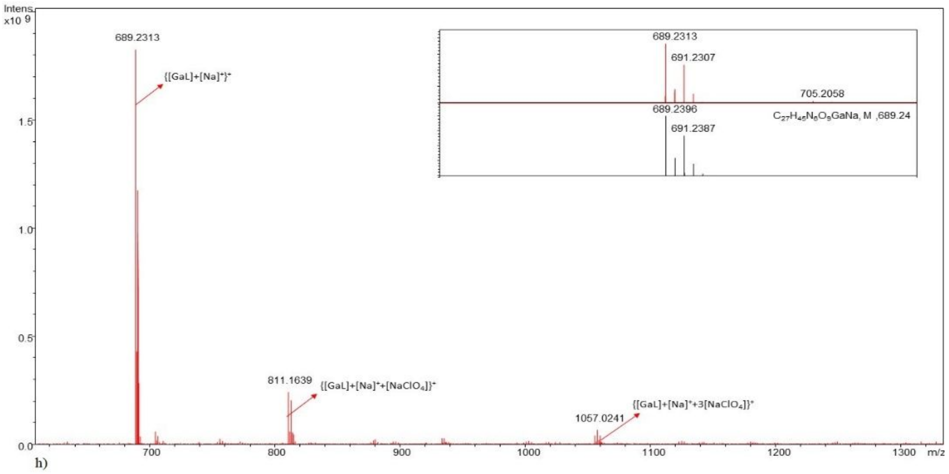
Intens. x10 9
689.2313
{[GaL]+[Na]+}+
689.2313
691.2307
705.2058
689.2396
C27H45N6O9GaNa, M ,689.24
691.2387
811.1639
{[GaL]+[Na]++[NaClO4]}+
{[GaL]+[Na]++3[NaClO4]}+
1057.0241
m/z
h)

i)

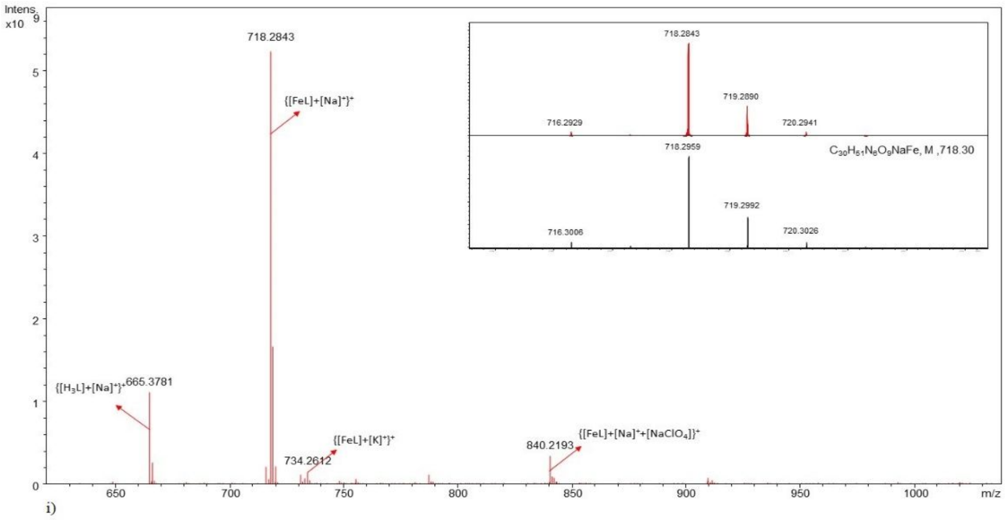

j)

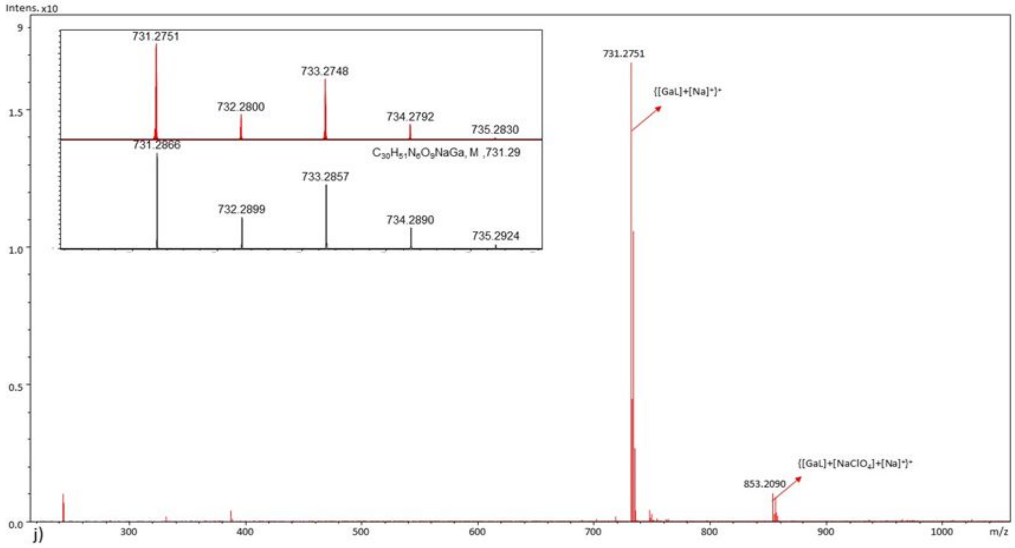

Figure S2. ESI-MS spectra for Fe(III)-FOX 2-2 (a), Ga(III)-FOX 2-2 (b), Fe(III)-FOX 2-3 (c), Ga(III)-FOX 2-3 (d), Fe(III)-FOX 2-4 (e), Ga(III)-FOX 2-4 (f), Fe(III)-FOX 2-5 (g), Ga(III)-FOX 2-5 (h), Fe(III)-FOX 3-5 (i), Ga(III)-FOX 3-5 (j), solution in metal to ligand molar ratio 1:1. The full spectra: expanded experimental (red) and simulated (black) spectra.

Table S2. Intensity maxima of the major Fe(III)-FOX complexes and adduct ions observed by ESI-MS

Fe(III) Complexes	Pseudomolcular ion	m/z experimental	m/z simulated
FOX 2-2	$\{[FeL] + [H]^+\}^+$	528.13	528.13
FOX 2-3	$\{[FeL] + [Na]^+\}^+$	592.15	592.16
	$\{[FeL] + [Na]^+ + [NaClO_4]\}^+$	714.09	714.09
	$\{[FeL] + [Na]^+ + 3[NaClO_4]\}^+$	957.95	957.97
FOX 2-4	$\{[FeL] + [Na]^+\}^+$	634.19	634.20
	$\{[FeL] + [Na]^+ + [NaClO_4]\}^+$	756.13	756.14
	$\{[FeL] + [Na]^+ + 3[NaClO_4]\}^+$	999.99	1000.01
FOX 2-5	$\{[FeL] + [Na]^+\}^+$	676.24	676.25
	$\{[FeL] + [Na]^+ + [NaClO_4]\}^+$	798.17	798.19
FOX 3-5	$\{[FeL] + [Na]^+\}^+$	718.29	718.27
	$\{[FeL] + [K]^+\}^+$	712.26	734.27
	$\{[FeL] + [Na]^+ + [NaClO_4]\}^+$	840.22	840.23

Conditions: $[L] = 1 \cdot 10^{-5}$ M, M:L 1:1, $MeOH/H_2O$: 50/50 w/w.

Table S3. Intensity maxima of the major Ga(III)-FOX complexes and adduct ions observed by ESI-MS

Ga(III) Complexes	Pseudomolcular ion	m/z experimental	m/z simulated
FOX 2-2	$\{[GaL] + [H]^+\}^+$	541.12	541.12
FOX 2-3	$\{[GaL] + [Na]^+\}^+$	605.14	605.14
	$\{[GaL] + [Na]^+ + [NaClO_4]\}^+$	727.07	727.08
	$\{[GaL] + [Na]^+ + 3[NaClO_4]\}^+$	927.93	927.96
FOX 2-4	$\{[GaL] + [Na]^+\}^+$	647.19	647.19
	$\{[GaL] + [Na]^+ + [NaClO_4]\}^+$	769.12	769.13
	$\{[GaL] + [Na]^+ + 3[NaClO_4]\}^+$	1014.97	1015.00
FOX 2-5	$\{[GaL] + [Na]^+\}^+$	689.23	689.24
	$\{[GaL] + [Na]^+ + [NaClO_4]\}^+$	811.16	811.18
	$\{[GaL] + [Na]^+ + 3[NaClO_4]\}^+$	1057.02	1057.05
FOX 3-5	$\{[GaL] + [Na]^+\}^+$	731.28	731.29

{[GaL] + [K]$^+$}$^+$	747.25	747.26
{[GaL] + [Na]$^+$ + [NaClO$_4$]}$^+$	853.21	853.22

Conditions: [L] = $1\cdot10^{-5}$ M, M:L 1:1, MeOH/H_2O: 50/50 w/w.

All peak assignments were based on the comparison between the calculated and experimental isotope patterns and correlate very well, with the species identified by the potentiometric and UV-Vis experiments. ESI–MS spectra collected for Fe(III)/Ga(III)-FOX analogs solutions in metal-to-ligand molar ratios of 1:1 and 1:2, confirmed the formation of monomeric complexes.

Ligand's protonation constants

The acid-base properties of investigated ligands were studied by potentiometric technique. The optimized logβ values defined by equations (S1) and (S2) were calculated using SUPERQUAD[1] or Hyperquad 2006[2] programs (Table S4). For the sake of simplicity charges are omitted in all chemical equilibria given here.

$$H_{n-1}L + H \leftrightarrow H_nL \qquad \text{(S1)}$$

$$K_n^H = [H_nL]/[H_{n-1}L][H] \qquad \text{(S2)}$$

Table S4. Protonation (logβ_H) constants for studied ligands[a]

Assingments	Compound					
	FOXE[b]	FOX 2-2	FOX 2-3	FOX 2-4	FOX 2-5	FOX 3-5
$L + H^+ \leftrightarrow HL$	9.89	9.97(1)	9.94(1)	9.93(1)	9.86(1)	10.19(1)
$HL + H^+ \leftrightarrow H_2L$	19.31	19.28(1)	19.27(1)	19.21(1)	19.09(1)	19.53(1)
$H_2L + H^+ \leftrightarrow H_3L$	27.94	27.62(2)	27.71(1)	27.64(1)	26.53(1)	27.79(1)

[a]Constants determined from potentiometric titrations. Conditions: [L] = $1\cdot10^{-3}$ M, T = 25°C, I = 0.1 M $NaClO_4$, [b] Ref [3]

Novel cyclic trihydroxamate analogs possess three hydroxamate moieties. For all analogs the protonation constants are within the range for hydroxamates known from literature, such as natural trihydroxamate FOXE.[3] All hydroxamate groups in FOXE are indistinguishable and because of the possible overlap of the dissociation processes, the pK values determined, cannot be ascribed unambiguously to either of the groups. Thus, to thoroughly determine the protonation processes of studied compounds, the pH-dependent NMR titrations would be

needed. However, such a detailed analysis is not necessary for the determination of stability of iron complexes and therefore was not performed.

Determination of stability constants

Complex formation equilibria with Fe(III)

During spectrophotometric titrations of Fe(III)–FOX systems, the color of the solutions changed from dark red in acidic pH to light yellow above pH 7. Spectral changes of LMCT bands (Fig. S3) were attributed to the formation of dihydroxamate $[FeHL]^{+}$ complex at acidic pH, and its further deprotonation and consecutive formation of trihydroxamate [FeL] complex, which persisted in solution up to pH ~ 8, where absorbance started to fade; at pH 11 we observed baseline elevation which was attributed to complex dissociation and Fe(III) hydroxide formation (data not shown). The formation of monohydroxamate $[FeH_2L]^{2+}$ species was not observed. Fe(III)-FOX 2-5 UV-Vis spectra were however different, with only one band present during whole titration (Fig. S3 g).

a)

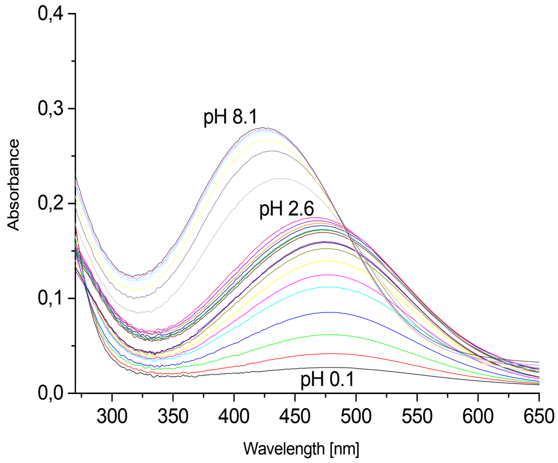
0,4
0,3
0,2
0,1
0,0
Absorbance
pH 8.1
pH 2.6
pH 0.1
300
350
400
450
500
550
600
650
Wavelength [nm]

b)

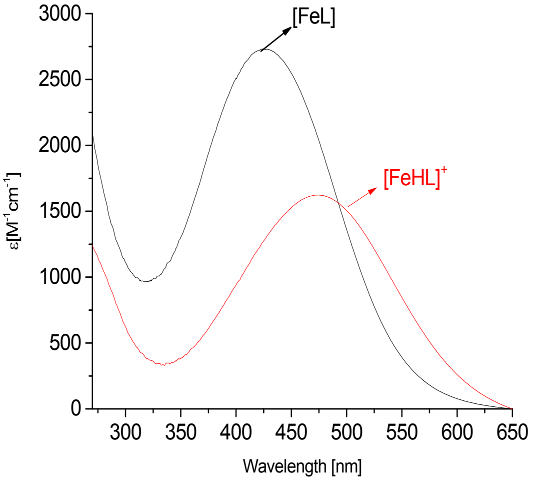
3000
2500
2000
1500
1000
500
0
ε[M-1cm-1]
[FeL]
[FeHL]+
300
350
400
450
500
550
600
650
Wavelength [nm]

c)

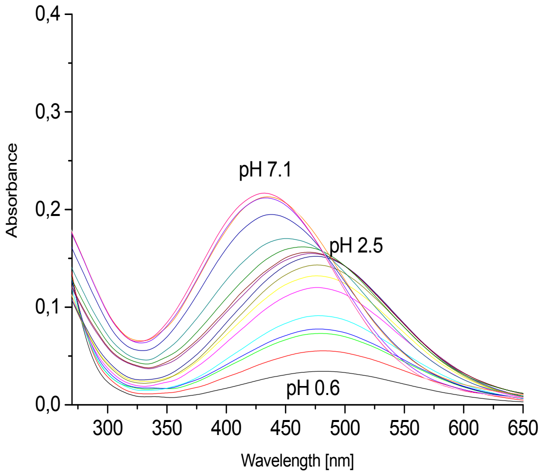
0,4
0,3
0,2
0,1
0,0
Absorbance
pH 7.1
pH 2.5
pH 0.6
300
350
400
450
500
550
600
650
Wavelength [nm]

d)

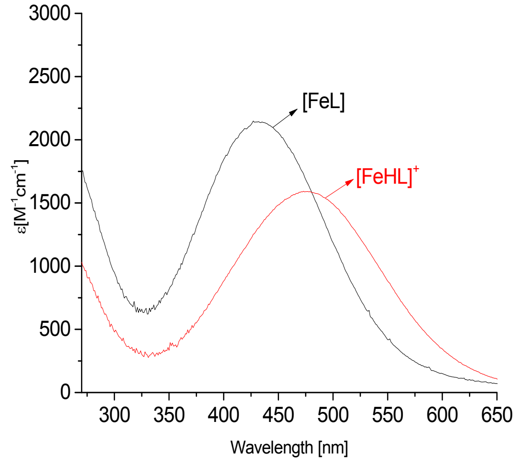
3000
2500
2000
1500
1000
500
0
ε[M-1cm-1]
[FeL]
[FeHL]+
300
350
400
450
500
550
600
650
Wavelength [nm]

e)

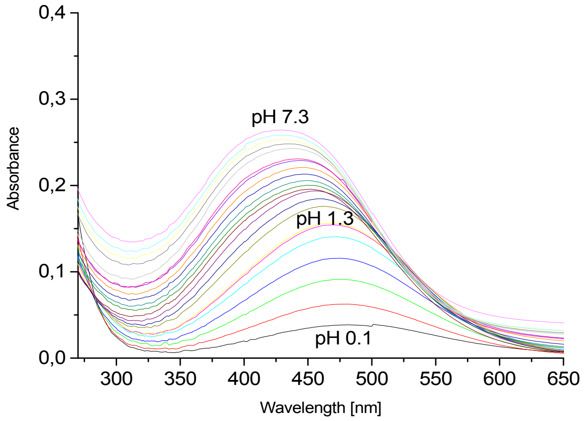
0,4
0,3
0,2
0,1
0,0
Absorbance
pH 7.3
pH 1.3
pH 0.1
300
350
400
450
500
550
600
650
Wavelength [nm]

f)

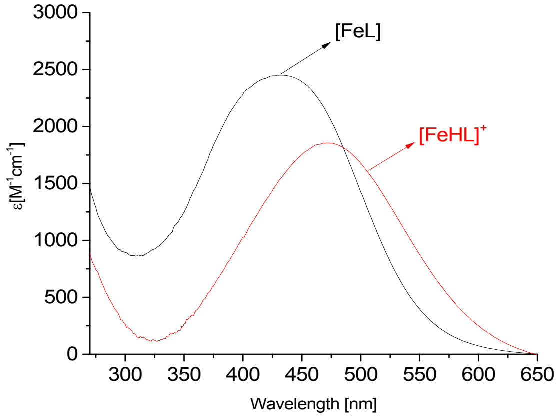
3000
2500
2000
1500
1000
500
0
ε[M-1cm-1]
[FeL]
[FeHL]+
300
350
400
450
500
550
600
650
Wavelength [nm]

g)

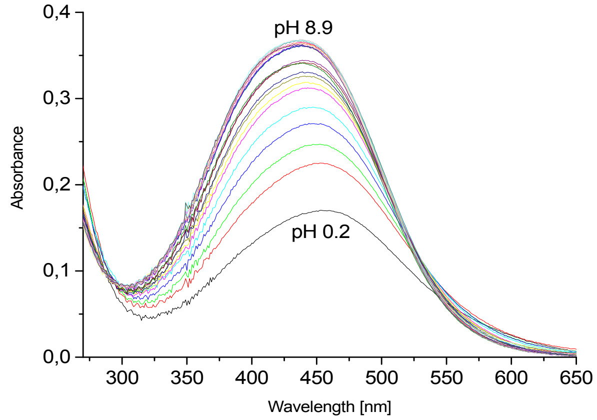

h)

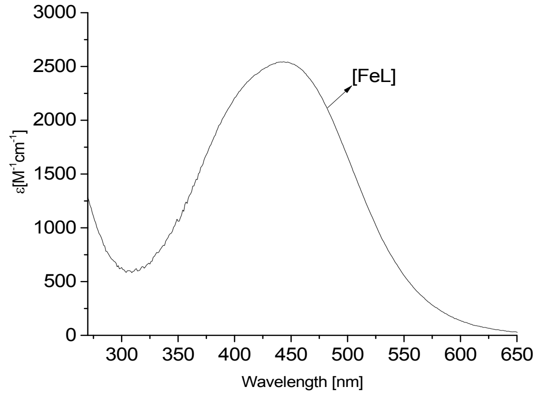

i)

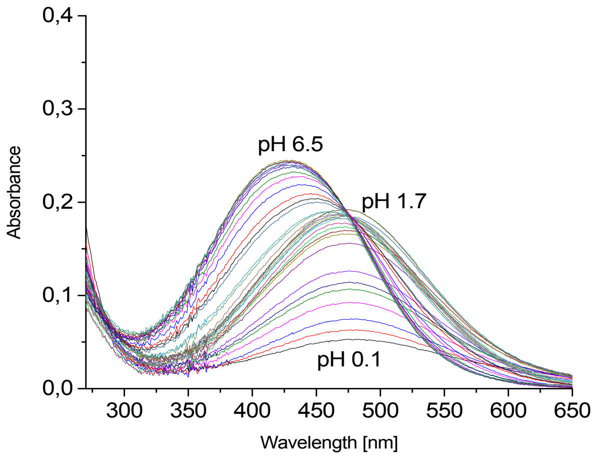

j)

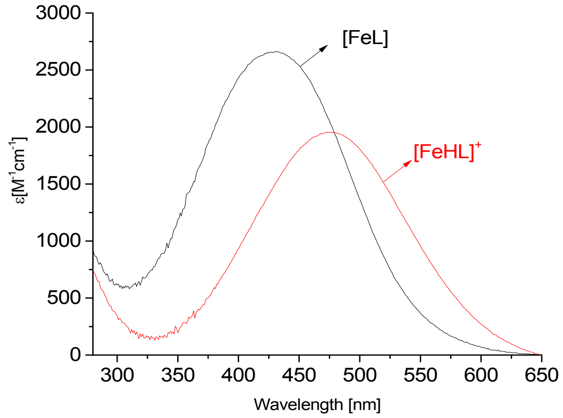

Figure S3. Spectroscopic data used to calculate constants (logβ) for studied complexes with Fe(III) ions and electronic spectra for Fe(III)-FOX 2-2 (a, b), Fe(III)-FOX 2-3 (c, d), Fe(III)-FOX 2-4 (e, f), Fe(III)-FOX 2-5 (g, h), Fe(III)-FOX 3-5 (i, j).
Conditions: [L] = $1\cdot10^{-4}$ M, M:L 1:1, T = 25°C, I = 0.1 M $NaClO_4$. Experimental errors: λ_{max} = ± 2nm, ε = ± 5%.

Stability constants gathered in Table S5 as logβ[FeH_mL_n], defined by equations (S3) and (S4), and spectroscopic characteristics of calculated spectra given in Table S6 were determined by refinement of the spectroscopic and potentiometric data.

$$Fe^{3+}_{(aq)} + L^{3-} \xleftrightarrow{K_{ML}} FeL \qquad (S3)$$

$$K_{ML} = \frac{[FeL]}{[Fe^{3+}_{(aq)}][L^{3-}]} \qquad (S4)$$

The uncertainties in the log$K$ values correspond to the added standard deviations in the cumulative constants.

Corresponding electronic spectra are presented in Fig. S3, while speciation plots are presented in Figure S7.

Table S5. Stability (logβ) constants for studied complexes with Fe(III) ions[a]

Assignments	Compound				
	FOX 2-2	FOX 2-3	FOX 2-4	FOX 2-5	FOX 3-5
$log\beta_{[FeHL]^+}$	30.64 (6)[c]	30.44 (5)[c]	30.83 (4)[c]	-	31.13[c]
$log\beta_{[FeL]}$	25.92 (8)[b]	27.22 (2)[b]	-	-	28.81 (1)[b]
$log\beta_{[FeL]}$	26.1[c]	26.70 (9)[c]	28.71 (7)[c]	31.32 (8)[c]	28.59 (3)[c]
pK_{NHOH}	4.72[b]	3.22[b]	-	-	2.32[b]
	4.54[c]	3.74[c]	2.12[c]	-	2.54[c]

[a]Constants determined from potentiometric[b] and UV-Vis[c] pH-dependent titrations. Conditions: [L] = $1\cdot10^{-3}$ M, M:L 1:1 for potentiometric assays, and $1\cdot10^{-4}$ M, M:L 1:1 for UV-Vis assays, T = 25°C, I = 0.1 M $NaClO_4$.

Values presented in Table S5 were calculated from data collected during measurements implementing two separate methods. As we can see above, this data correlates very well and further confirms our calculations.

Table S6. Spectroscopic parameters for studied complexes[a]

Complex form	Analog									
	FOX 2-2		FOX 2-3		FOX 2-4		FOX 2-5		FOX 3-5	
	λ_{max} [nm]	ε [$M^{-1}cm^{-1}$]	λ_{max} [nm]	ε [$M^{-1}cm^{-1}$]	λ_{max} [nm]	ε [$M^{-1}cm^{-1}$]	λ_{max} [nm]	ε [$M^{-1}cm^{-1}$]	λ_{max} [nm]	ε [$M^{-1}cm^{-1}$]
$[FeHL]^+$	476	1620	478	1590	473	1857	-	-	476	1950
[FeL]	426	2730	432	2140	430	2450	440	2540	430	2660

[a]Conditions: [L] = $5\cdot10^{-5}$ M, M:L 1:1, T = 25°C, I = 0.1 M $NaClO_4$. Experimental errors: λ_{max} = ± 2nm, ε = ± 5%.

The spectrophotometric competition experiments with EDTA

The spectrophotometric competition experiments with EDTA were performed at fixed pH 7.0, where fully formed complexes of Fe(III)-FOXE analogs were formed, characterized by strong bands at 430-440 nm. Those LMCT bands were gradually silenced after addition of fixed amount of EDTA ligand, from 0-10 molar equivalent for FOX 2-5 and 0-1.2 molar equivalent for rest of the ligands (Fig. S4).

a)

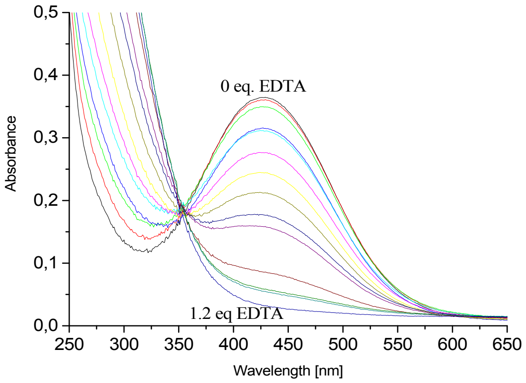

b)

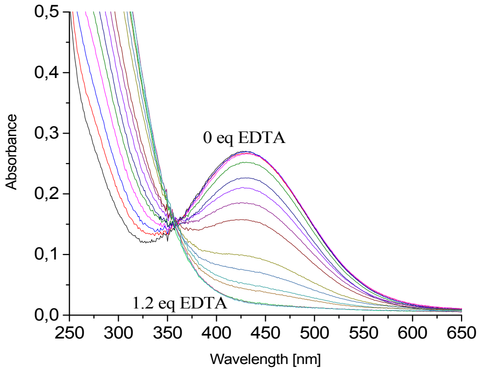

c)

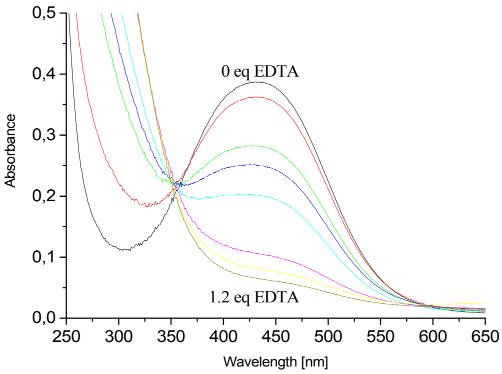

d)

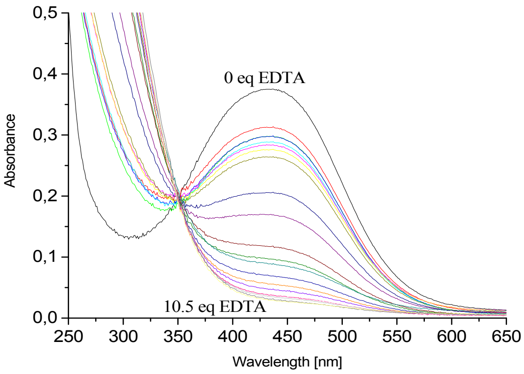

e)

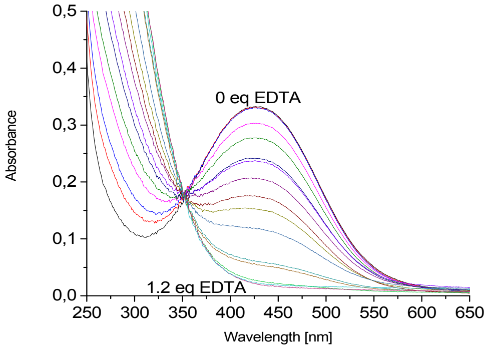

Figure S4. UV-Vis spectra of competition reaction of Fe(III)-FOXE analog complexes vs EDTA for FOX 2-2 (a), FOX 2-3 (b), FOX 2-4 (c), FOX 2-5 (d), and FOX 3-5 (e).
Conditions: [L] = $1.5\cdot10^{-4}$ M, $T = 25°C$, $I = 0.1$ M $NaClO_4$.

The stability of ferric ion complexes with EDTA is of sufficient strength ($\log\beta_{Fe(III)EDTA} = 10^{25.0}$) to compete effectively with the tris-hydroxamates for ferric ion at intermediate pH values (5-8) if an excess of EDTA is used. The competition equilibrium is described by equations (S5) and (S6).

$$Fe^{III}L + EDTA \xleftrightarrow{K} Fe^{III}EDTA + L \qquad \text{(S5)}$$

$$K = \frac{[Fe^{III}EDTA][L]}{[Fe^{III}L][EDTA]} = \frac{\beta_{110}^{FeEDTA}}{\beta_{110}^{Fe(III)L}} \qquad \text{(S6)}$$

The competition data were refined to obtain the overall Fe(III)-FOX binding constant $\log\beta_{FeL}$ (Table S7) for analogs using a model involving two ligands and one metal. The protonation constants and Fe(III) formation constants for EDTA[4] and the protonation constants of the analogs (The acid-base properties of investigated ligands were studied by potentiometric technique. The optimized $\log\beta$ values defined by equations (S1) and (S2) were calculated using SUPERQUAD[1] or Hyperquad 2006[2] programs (Table S4). For the sake of simplicity charges are omitted in all chemical equilibria given here.

$$H_{n-1}L + H \leftrightarrow H_nL \qquad \text{(S1)}$$

$$K_n^H = [H_nL]/[H_{n-1}L][H] \qquad \text{(S2)}$$

Table S4), were used as fixed parameters during data analysis.

The concentration of Fe(III)-analog complex for each derivative, [FeL], was calculated from the absorbance maximum at around 420 nm (where FeL is the only light-absorbing species) and previously determined extinction coefficients. The concentrations of other species in equation (S6) were calculated from mass balance equations using the experimental pH values:

$$[Fe]_{tot} = \alpha_{FeL}[FeL] + \alpha_{FeEDTA}[FeEDTA] \quad (S7)^a$$

$$[L]_{tot} = \alpha_{FeL}[FeL] + \alpha_{L}[L] \quad (S8)^a$$

$$[EDTA]_{tot} = \alpha_{FeEDTA}[FeEDTA] + \alpha_{EDTA}[EDTA] \quad (S9)^a$$

[a]α is the usual Ringbom's coefficient.[5]

Table S7. Stability ($\log\beta_{FeL}$) constants for studied complexes with Fe(III) ions[a]

Assignments	Compound				
	FOX 2-2	FOX 2-3	FOX 2-4	FOX 2-5	FOX 3-5
$log\beta_{[FeL]}$	26.57 (4)	26.55 (3)	27.13 (1)	28.81 (1)	28.38 (1)

[a]Constants determined from EDTA competition studies. Conditions: [L] = $1.5 \cdot 10^{-4}$ M, M:L ratio 1:1, pH = 7, T = 25°C, I = 0.1 M $NaClO_4$.

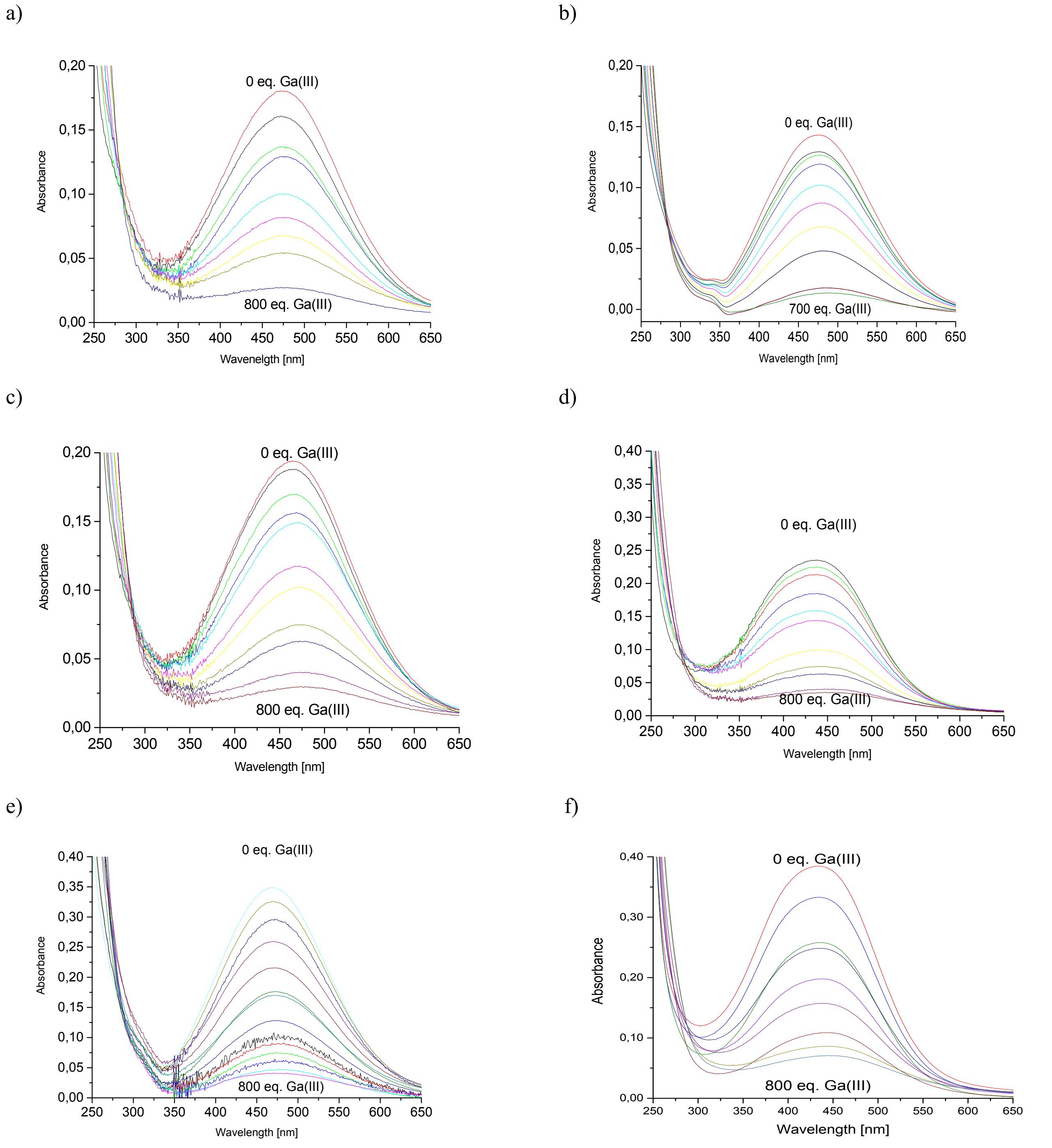

Figure S5. Spectroscopic data used to calculate (logβ) constants for studied complexes with Ga(III) ions for FOX 2-2 (a), FOX 2-3 (b), FOX 2-4 (c), FOX 2-5 (d), FOX 3-5 (e) and FOXE (f).

Conditions: a), b), d) [L] = $1 \cdot 10^{-4}$ M, M:L ratio 1:1, pH = 2; c), e) [L] = $1 \cdot 10^{-4}$ M, M:L ratio 1:1, pH = 1.4; pH = 1.5; f) [L] = $1.5 \cdot 10^{-4}$ M, M:L ratio 1:1, pH = 2; T = 25°C, I = 0.1 M $NaClO_4$.

Table S8. Stability (logβ) constants for studied complexes with Ga(III) ions[a]

Assignments	Compound					
	FOX 2-2	FOX 2-3	FOX 2-4	FOX 2-5	FOX 3-5	FOXE
$log\beta_{[GaHL]^+}$	28.48 (4)[c]	28.30 (4)[c]	28.84 (5)[c]	-	29.45 (1)[c]	-
$log\beta_{[GaL]}$	26.52 (5)[b]	26.02 (1)[b]	26.28 (1)[b]	29.52 (6)[c]	26.58 (4)[b]	29.79 (1)
pK_{NHOH}	3.25	2.28	2.56	-	2.87	-

[a]Constants determined from potentiometric[b] and UV-Vis[c] pH-dependent titrations. Conditions: [L] = $1 \cdot 10^{-3}$ M, M:L 1:1 for potentiometric assays and [L] = $1 \cdot 10^{-4}$ M, M:L 1:1 for competition UV-Vis titrations, and [L] = $5 \cdot 10^{-5}$ M, M:L 1:1 for UV-Vis pH-metric titration assays; T = 25°C, I = 0.1 M $NaClO_4$.

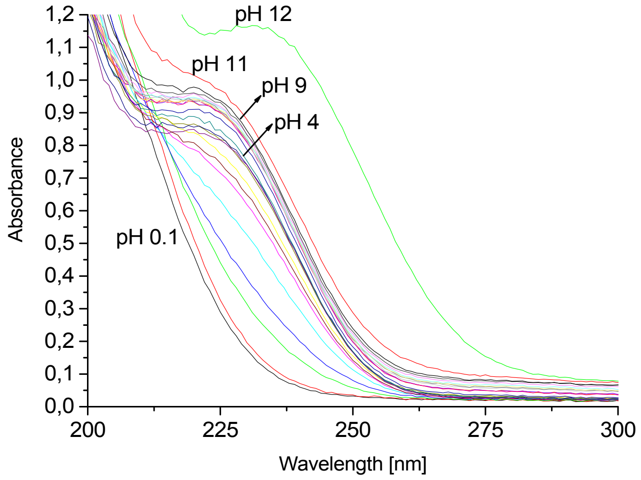

Figure S6. pH-dependent spectroscopic titration of Ga(III)-FOX 2-5 analog complex.
Conditions: [L] = $5 \cdot 10^{-5}$ M, M:L ratio 1:1, pH range 0.1-11.9, T = 25°C, I = 0.1 M $NaClO_4$.

a) b)

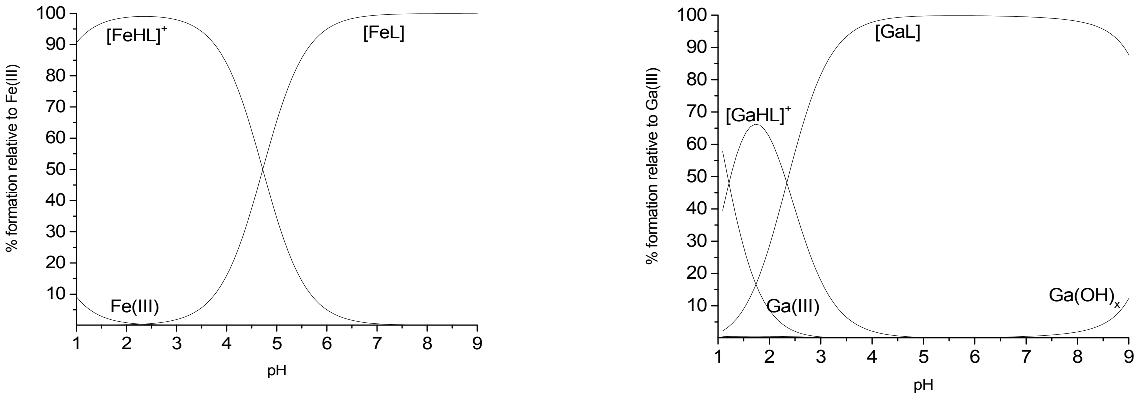

c)

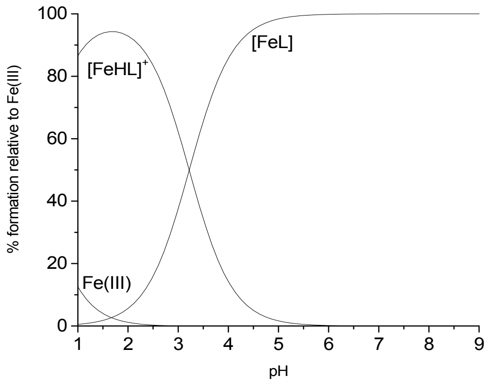
[FeHL]+
[FeL]
Fe(III)
% formation relative to Fe(III)
pH

d)

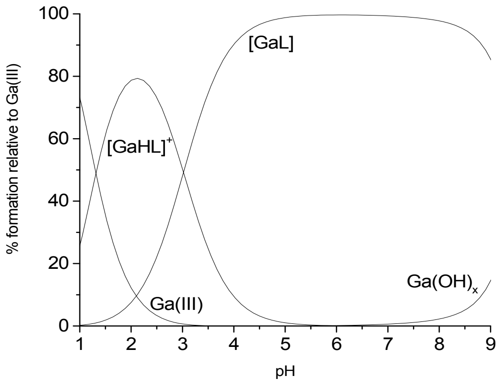
[GaL]
[GaHL]+
Ga(III)
Ga(OH)x
% formation relative to Ga(III)
pH

e)

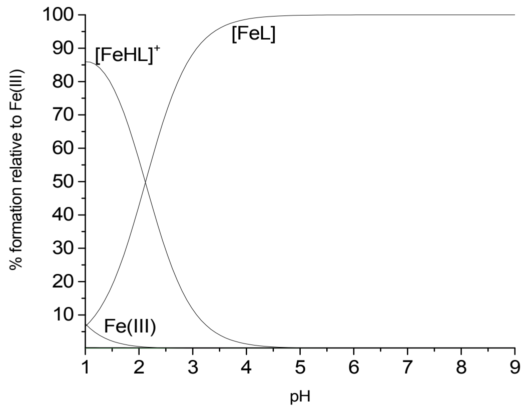
[FeHL]+
[FeL]
Fe(III)
% formation relative to Fe(III)
pH

f)

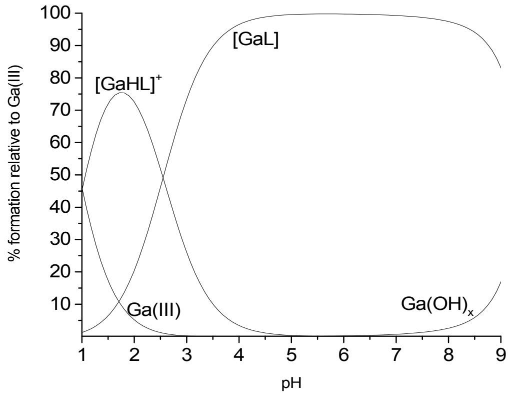
[GaL]
[GaHL]+
Ga(III)
Ga(OH)x
% formation relative to Ga(III)
pH

g)

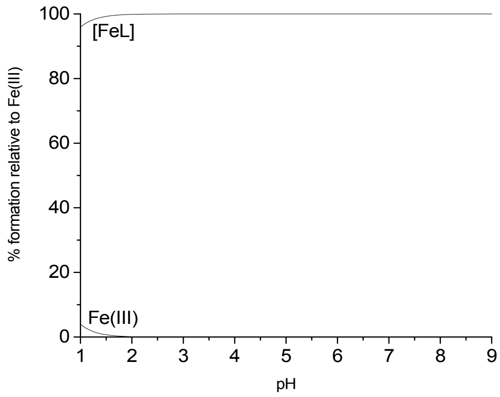
[FeL]
Fe(III)
% formation relative to Fe(III)
pH

h)

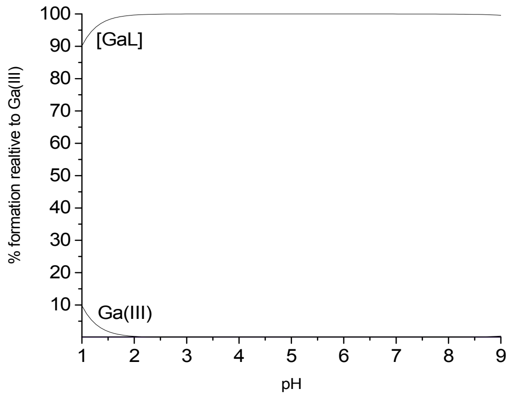
[GaL]
Ga(III)
% formation realtive to Ga(III)
pH

i)

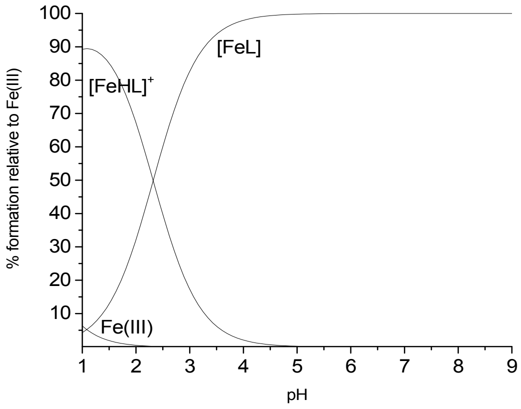

j)

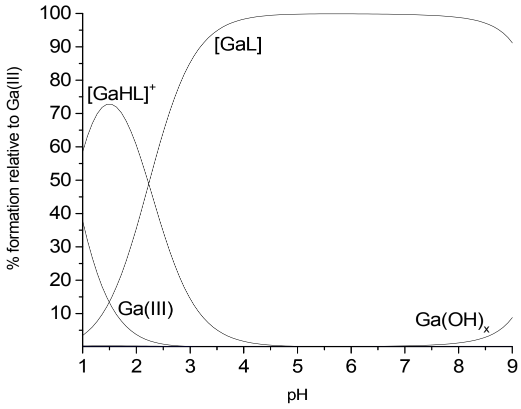

Figure S7. Species distribution for studied complexes with Fe(III) and Ga(III) ions[a]: a) Fe(III)-FOX 2-2, c) Fe(III)-FOX 2-3, e) Fe(III)-FOX 2-4, g) Fe(III)-FOX 2-5, h) Fe(III)-FOX 3-5 b) Ga(III)-FOX 2-2, d) Ga(III)-FOX 2-3, f) Ga(III)-FOX 2-4, h) Ga(III)-FOX 2-5, j) Ga(III)-FOX 3-5

[a]Plots calculated for conditions: [L] = $1\cdot10^{-3}$, [Fe(III)] = $1\cdot10^{-3}$

Complex formation equilibria with Fe(II)

a)

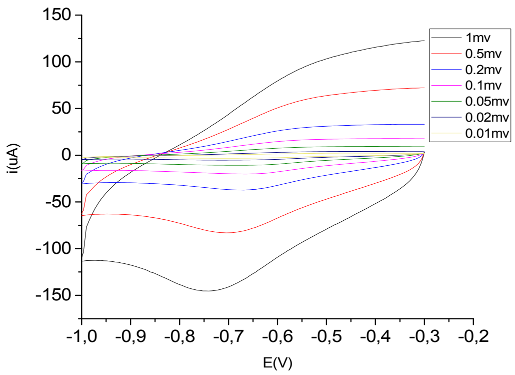

b)

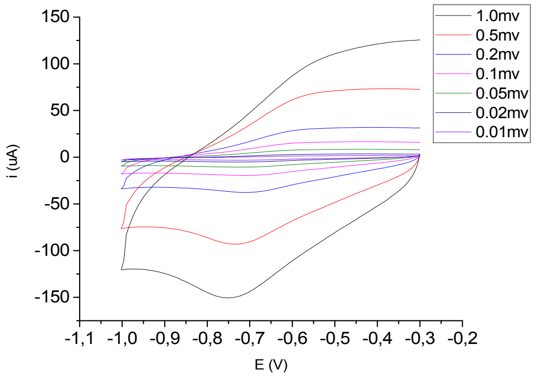

c)

d)

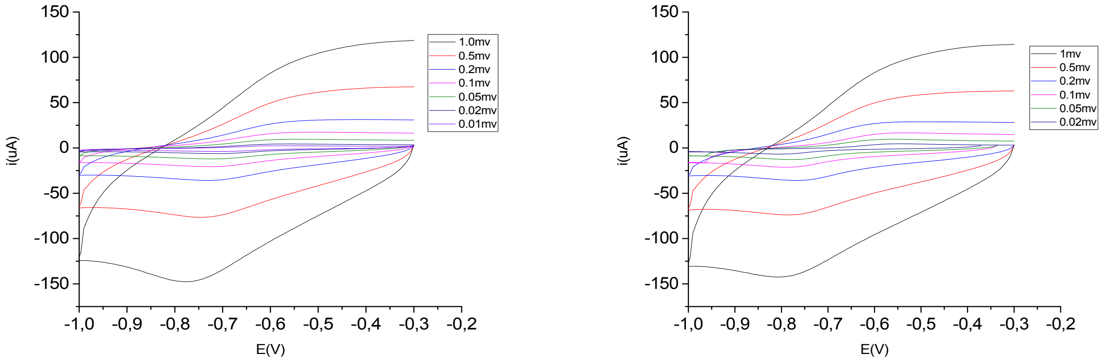

e)

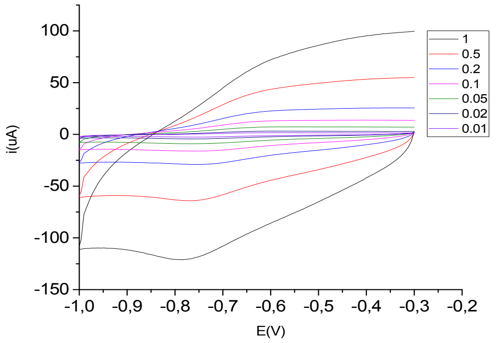

Figure S8. CV voltammograms used to calculate (logβ) constants for studied complexes[a] with Fe(II) ions: a) FOX 2-2, b) Fe-FOX 2-3, c) FOX 2-4, d) FOX 2-5, e) FOX 3-5

[a]Conditions: [Fe(III)] = $2\cdot10^{-3}$ M, M:L ratio 1:1, pH = 7, T = 25°C, I = 0.1M $NaClO_4$ on glass-carbon working electrode.

For each complex the stability constant for Fe(II)-analog was calculated using eq. S10

$$E^0{}_{complex} - E_{aq}{}^0 = -59.15\log\left(\frac{\beta^{III}}{\beta^{II}}\right) \text{ (S10)}$$

Where $E_{aq}{}^0 = 771\ mV$ and $NHE = 222\ mV$.

Table S9. $E_{1/2}$ and stability (logβ) constants for studied complexes with Fe(II) ions[a]

Assignments	Compound				
	FOX 2-2	FOX 2-3	FOX 2-4	FOX 2-5	FOX 3-5
E°complex [V]	-369	-432	-426	-452	-416

$\log\beta_{[Fe(II)L]^-}$	7.45	7.54	9.12	11.29	8.48

[a]Constants determined from CV measurements. Conditions: [L] = $1\cdot10^{-3}$ M, M:L ratio 1:1, pH = 7, T = 25°C, I = 0.1 M $NaClO_4$.

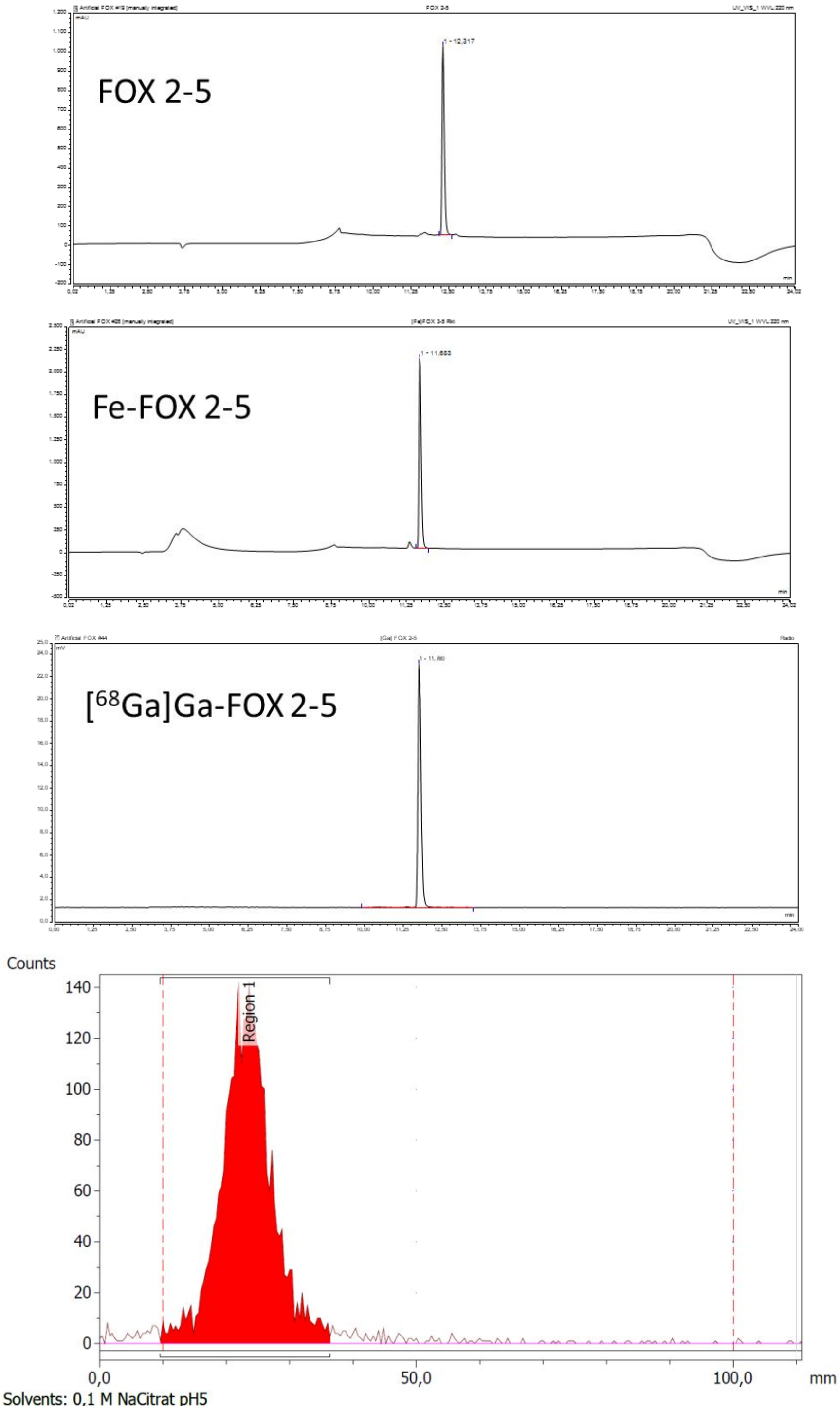

Fig. S10. Representative UV-HPLC chromatograms of FOX-2-5 and Fe-FOX-2-5 (UV 220 nm), radio-HPLC and radio-TLC chromatograms of [^{68}Ga]Ga-FOX 2-5, from top to bottom.

Table S10. Radiochemical Purity (RCP) of [^{68}Ga]GaFOX analogs as determined by SPE, HPLC and TLC

Analog	SPE[a]			HPLC			TLC		
	n	RCP	sd	n	RCP	sd	n	RCP	sd
FOX 2-2	5	75.1	7.9	3	51.1	12.3	5	13.8	5.4
FOX 2-3	5	52.5	6.8	3	13.4	4.1	5	13.8	5.4
FOX 2-4	5	94.2	2.3	5	83.4	2.4	5	15.1	8.8
FOX 2-5	n.d.	n.d.	n.d.	5	99.3	0.4	5	99.1	0.9
FOX 2-6	3	>90[b]		4	90.6	5.9	4	90.2	8.0
FOX 3-5	5	86.5	24.3	3	80.8	3.1	5	n.a.	n.a.

[a]RCP calculated as percentage of bound (eluted in the ethanol fraction) over total activity eluted;

[b]No exact calculation was made, as in one case RCP exceeded 100% due to geometry effects; n.d.: not determined (as RCP was exceeding 95% in other analysis), n.a.: not applicable due to insufficient separation

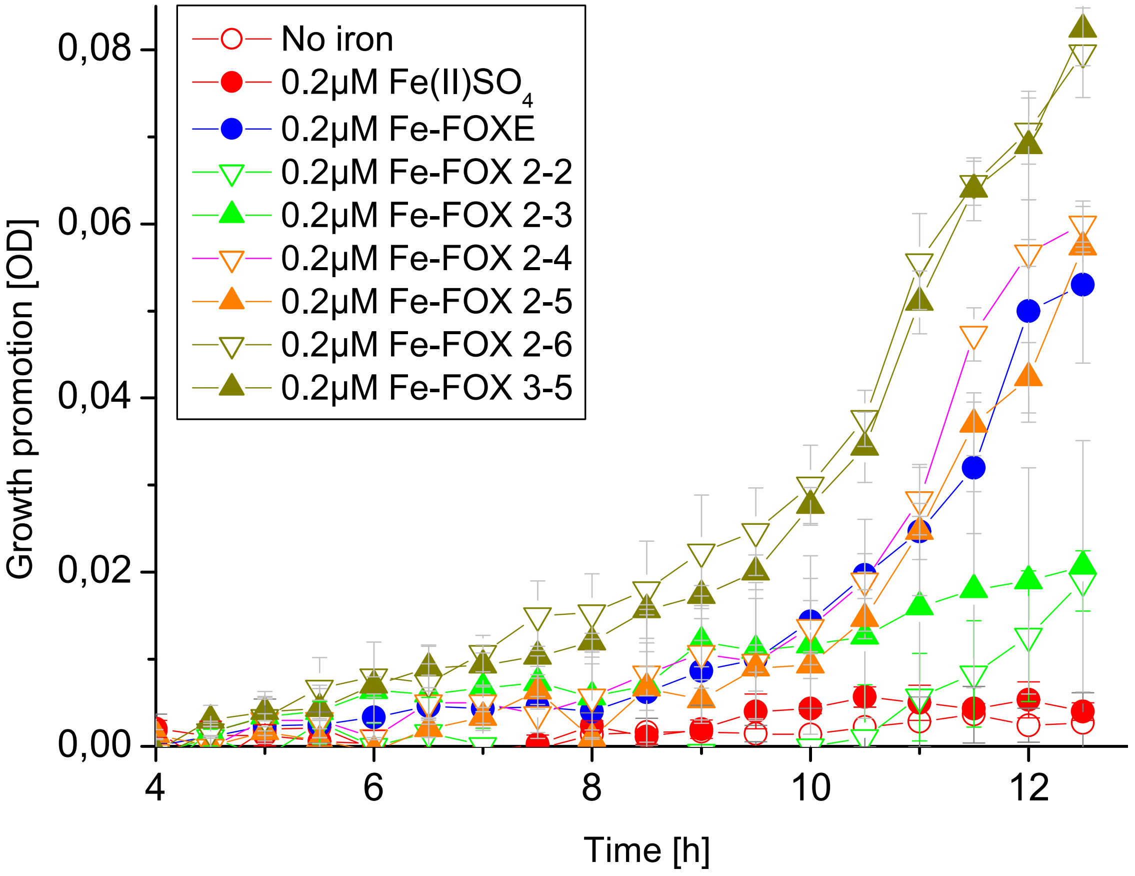

Fig. S11. Growth promotion of Fe-FOX derivatives in comparison with Fe-FOXE, Fe(II)-SO_4 and no iron supply.

References

1. Gans, P.; Sabatini, A.; Vacca, A. SUPERQUAD: an improved general program for computation of formation constants from potentiometric data. *Journal of the Chemical Society-Dalton Transactions* **1985,** (6), 1195-1200, DOI: 10.1039/dt9850001195.
2. Alderighi, L.; Gans, P.; Ienco, A.; Peters, D.; Sabatini, A.; Vacca, A. Hyperquad simulation and speciation (HySS): a utility program for the investigation of equilibria involving soluble and partially soluble species. *Coordination Chemistry Reviews* **1999,** (184), 311-318, DOI: 10.1016/s0010-8545(98)00260-4.
3. Smith, R.; Martell, A. *Hydroxamic Acids*. Springer, Boston, MA: 1989; 6, 416-420.
4. Pettit, L. D.; Powell, H. K., The IUPAC Stability Constants Database Academic Software. In Timble, York, UK: 2001; (www.acadsoft.co.uk, version 5.6).
5. Ringbom, A. Complexation in analytical chemistry : a guide for the critical selection of analytical methods based on complexation reactions. Interscience: New York, 1963.
